# Charge transfer and X-ray absorption investigations in aluminium and copper co-doped zinc oxide nanostructure for perovskite solar cell electrodes

**DOI:** 10.1038/s41598-023-37754-1

**Published:** 2023-07-04

**Authors:** Mandeep Kaur, Sanjeev Gautam, Keun Hwa Chae, Wantana Klysubun, Navdeep Goyal

**Affiliations:** 1 Advanced Functional Materials Laboratory, Dr. S. S. Bhatnagar University Institute of Chemical Engineering & Technology, Panjab University, Chandigarh, 160014 India; 2 Advanced Analysis & Data Center, Korea Institute of Science and Technology, Seoul, 02792 South Korea; 3 Synchrotron Light Research Institute, Nakhon Ratchasima, 30000 Thailand; 4 Department of Physics, Panjab University, Chandigarh, 160014 India

**Keywords:** Materials chemistry, Physical chemistry, Chemical synthesis

## Abstract

This study explores influence of charge transfer and X-ray absorption characteristics in aluminum (Al) and copper (Cu) co-doped zinc oxide (ZnO) nanostructures for perovskite solar cell electrodes. Sol-gel technique was employed to synthesize the nanostructures, and their optical and morphological properties were investigated. X-ray diffraction (XRD) analysis confirmed high crystallinity and also single-phase composition of all the samples, particularly up to 5% Al co-doping. Field emission scanning electron microscopy (FESEM) exhibited the formation of pseudo-hexagonal wurtzite nanostructure and the transition to nanorods at 5% Al co-doping. Diffuse reflectance spectroscopy indicated a reduction in the optical band gap of co-doped zinc oxide from 3.11 to 2.9 eV with increasing Al doping. Photoluminescence spectra (PL) exhibited a decrease in peak intensity, suggesting enhanced conductivity in ZnO, also confirmed from I-V measurements. Near-edge X-ray absorption fine structure (NEXAFS) analysis depicts that charge transfer from Al to oxygen (O) species enhanced the photosensing properties of the nanostructure, which was supported by FESEM micrographs and PL spectra. Furthermore, the study discovered that 5% Al co-doping significantly reduced the density of emission defects (deep-level) in Cu–ZnO nanostructure. These findings highlight the potential of Cu and Al co-doped ZnO materials for perovskite solar cell electrodes, as their improved optical and morphological properties resulting from charge transfer could enhance device performance. The investigation of charge transfer and X-ray absorption characteristics provides valuable insights into the underlying mechanisms and behaviors of the co-doped ZnO nanostructures. However, further research is required to delve into the intricate hybridization resulting from charge transfer and explore the broader impact of co-doping on other properties of the nanostructures, enabling a comprehensive understanding of their potential applications in perovskite solar cells.

## Introduction

Photodetectors comprising organic/inorganic nanostructures have found numerous applications in areas such as organs-on-chip^1,2^, wearable prosthetics^3,4^, and noninvasive pathology^5^. As a consequence, there is a growing interest in high-performance photodetectors using semiconductor oxides for various applications, including water purification, space communications, and pollution monitoring^6^.

Among the various oxide semiconductors used in photodetector fabrication, zinc oxide (ZnO) has gained outstanding attraction due to wide band gap (3.37 eV), high optical transmittance, and high electron mobility, which are necessary for efficient electron transport and for reduction recombination rates^7,8^. In recent years, much research has been done on photodetectors by growing zinc oxide nanorods via sol-gel technique^9,10^, and efforts to control the structure and morphology of ZnO to enhance its performance by manipulating growth parameters^11^.

In this context, the co-doping of Cu and Al in the ZnO system has been analyzed as a means to improve photodetector performance^12^. Additionally, Al and Cu-doped ZnO have shown promise as electrode materials for perovskite solar cells, offering high electrical conductivity, excellent optical properties, and good stability^13^. When used as front electrodes, Al and Cu-doped ZnO can enhance light absorption and cell efficiency due to their high optical transparency and low reflectivity, enabling increased light penetration and absorption by the active layer. Moreover, they act as effective barrier layers, preventing moisture and oxygen diffusion into the solar cell, which can degrade performance and reduce the cell’s lifespan. Therefore, doping ZnO with Al or Cu enhances electrical conductivity and stability compared to undoped counterparts by increasing carrier concentration and reducing defects in the ZnO lattice. Consequently, Al and Cu-doped ZnO exhibit considerable potential as electrode materials for perovskite solar cells, with further optimization achievable through material engineering and device design^14^.

Additionally, Zhou et al. demonstrated that mesoporous carbon electrode structures can improve the photovoltaic performance of perovskites^15^, while ion implantation techniques have shown promise in enhancing efficiency, conductivity, and reducing carrier recombination rates^16^. Photodetectors have gained popularity for their applications in various industries, such as fuel cells (hydrogen gas detection)^17^, mining (methane detection)^18^, automobiles (NO$_2$ detection from vehicle exhaust)^19^, oil refineries (hydrocarbon detection)^20^, and fertilizer production (ammonia detection)^21^. Additionally, Al-doped Cu–ZnO samples have explored for their potential use in hydrogen generation, solar cells, water splitting, light-emitting diodes, and plasmonic applications^22–25^. Researchers have investigated the influence of metal co-doping on defects (intrinsic), electrical, and optical properties by changing Al doping in the Cu–ZnO system^26^.

Doping involves introducing impurities into a pure material to modify its properties. The doping atom, whether it behaves as an electron acceptor or donor, induces electron delocalization within doped material. In the case of ZnO, trivalent Al dopants create an additional band in the middle of lowest unoccupied molecular orbital (LUMO) and the highest occupied molecular orbital (HOMO), functioning as an electron bridge for enhancing electron excitation, and hence reducing recombination rates. The doping process in ZnO with trivalent Al results in *p*-type doping, where three valence electrons from Al attract one electron from the ZnO lattice, leaving holes in ZnO valence band. This introduces electron delocalization within ZnO system and hence the positively charged hole facilitates electron movement creating a cyclic electron flow^27^.

In ZnO crystals, the two electrons from the 4$s^2$ orbital of Zn interact with the four electrons from 2$p^6$ orbital of oxygen, filling up 2$p^6$ orbital of oxygen. As a result, ZnO possesses an unoccupied 4$s^2$ Zn orbital and a fully occupied 2$p^6$ O orbital, making it *n*-type semiconductor (intrinsic) because of the ionization of excess electrons in 4$s^2$ orbital of Zn. The *n*-type behavior of zinc oxide is believed to arise from presence of oxygen vacancy or Zn interstitial, although exact cause of the *n*-type conductivity is still a matter of debate^28,29^. The accumulation of *n*-type charge carriers and rapid recombination rates in pristine ZnO materials lead to photosensing phenomena. Doping of zinc oxide system with metals like Al, Cu, Ni, and Mn can address this issue and yield optoelectronic materials with improved qualities for photosensor applications^30,31^. The substitution of Al to Cu–ZnO enhances the electronic characteristics through exchange interactions, facilitating charge transfer from the HOMO to the LUMO^32,33^. While significant research has been conducted on pristine ZnO in recent years, the exploration of photodetector device fabrication using Al/Cu co-doped zinc oxide remains relatively limited.

In this study, preparation of zinc oxide and co-doped ZnO at different Cu, Al concentrations has been done using sol-gel technique. Structural, morphological and optical properties are used to make a analysis of comparative study using various systemetic characterization techniques, such as XRD, FESEM, PL, XRD, XAS and UV. Al$^{3+}$ ions have been used to study enhanced photosensing properties and charge-transfer effect. The impact of Al incorporated Cu–ZnO structure on morphological growth of nanostructure is also investigated. The doping with Al improves the quality of crystallinity of Cu–ZnO nanoparticles and remarkably suppresses density of deep-level emission defect in Cu–ZnO revealed by X-ray diffraction, photoluminescence, and X-ray absorption spectroscopy. The correlation between near edge X-ray absorption fine structures (NEXAFS) and Field Emission Scanning Electron Microscopy (FESEM) micrographs for Al co-doped Cu–ZnO nanostructures is also been studied. The outstanding performance in photodetectors has been observed for Al-doped Cu–ZnO system.

## Materials and methods

The pristine ZnO and Al$_x$Cu$_{y}$Zn$_{1-x-y}$O (*y*=0.00, 0.005, *x*=0.00, 0.005, 0.01, 0.03, and 0.05) samples were prepared using sol-gel approach. Zinc acetate dihydrate and copper acetate monohydrate, along with aluminum chloride hexahydrate, served as the source of zinc and dopants, respectively. All chemical reagents, including copper acetate monohydrate, sodium hydroxide (NaOH), aluminum chloride hexahydrate, and zinc acetate dihydrate, were obtained from Sigma-Aldrich.

In the typical process, the aforementioned reagents (excluding NaOH) are dissolved in distilled water according to their stoichiometry. NaOH solution is dissolved in de-ionized water and it is added to solution dropwise with constant stirring by using temperature of 60 $^\circ$C, maintaining a pH of approximately 9-10, until milky precipitates formed. The obtained precipitates were centrifuged at 6000 rpm for 2 minutes, then washed/rinsed using de-ionized water/ethanol. Subsequently, they were dried for 7 hours in an oven at 100 $^\circ$C and then annealed at 400 $^\circ$C by using muffle furnace for 12 hours. Finally, the samples were ground to a fine powder by utilizing a mortar and pestle. Using a similar method, samples with different concentrations of copper and aluminum were prepared. The synthesized samples were ZnO, Zn$_{0.995}$Cu$_{0.005}$O, Zn$_{0.995}$Al$_{0.005}$O, Zn$_{0.99}$Cu$_{0.005}$Al$_{0.005}$O, Zn$_{0.985}$Cu$_{0.005}$Al$_{0.01}$O, Zn$_{0.965}$Cu$_{0.005}$Al$_{0.03}$O, and Zn$_{0.945}$Cu$_{0.005}$Al$_{0.05}$O. For further discussions in the text, these samples were referred to as Pr, Cu(0.5), Al(0.5), CuAl(0.5), Al(1), Al(3), and Al(5), respectively.

The crystalline behavior of the synthesized samples was confirmed using X-ray diffraction (PANalytical Model-X’pert pro, Netherlands) with Cu K$_\alpha$ radiation ($\lambda$=0.154 nm). The diffraction patterns were recorded from 20$^\circ$ to 80$^\circ$ Bragg’s angle with a step size of 1$^\circ$ per minute to obtain fine structural resolution so that it can be used for Reitveld’s refinement.

Morphological and structural studies were conducted using FE-SEM images captured by a Hitachi (Japan) Model-SU 8010 series (resolution of 1 nm) and a landing voltage of about 1 KV. The band gaps of different samples were calculated using UV-DRS spectroscopy, and PL analysis was performed to analyze the fluorescence nature of the synthesized samples. The I-V characteristics for photo-sensing investigations were performed using the electrochemical workstation M204 (Metrohm AutoLab) at the central facility lab, Dr. SSB UICET, Panjab University, Chandigarh. Near-edge X-ray absorption fine-structure (NEXAFS) evaluation for O K-edge and Zn L-edge was carried out on the samples at the 10D (XAS-KIST) beamline of the Pohang Accelerator Laboratory (KIST-PAL beamline) in South Korea. Cu K-edge NEXAFS data were collected in transmission mode at BL8, Synchrotron Light Research Institute (SLRI), Thailand.

## Results and discussion

### X-ray diffraction study

**Figure 1:**
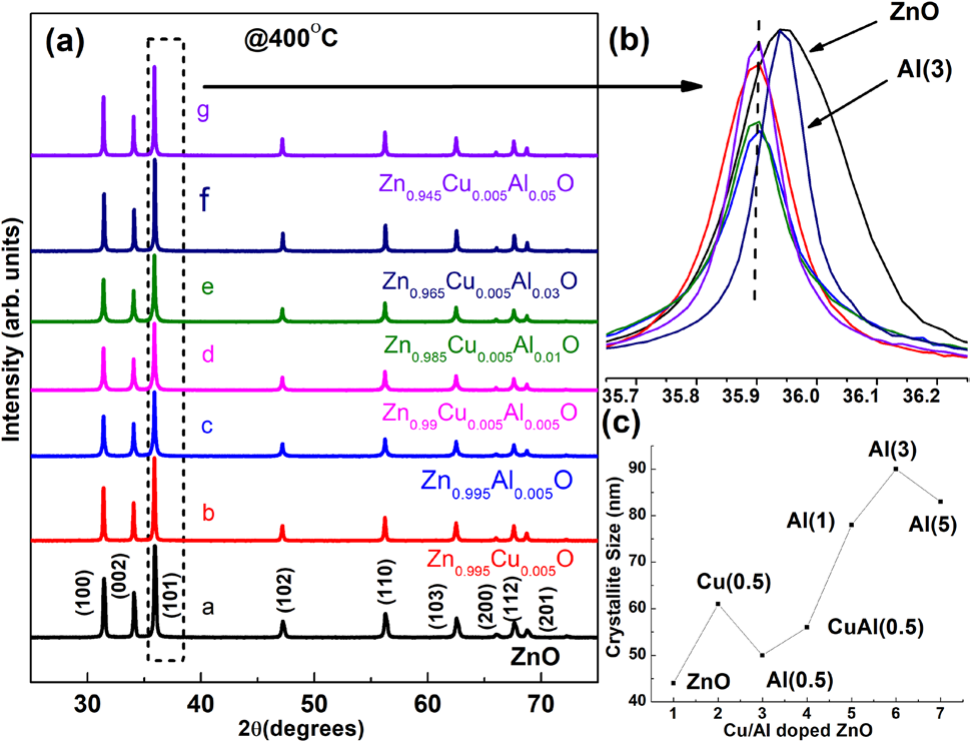
(**a**) Comparison of XRD patterns of Al$_{\text {x}}$Cu$_{\text {y}}$Zn$_{\text {1-x-y}}$O (y=0.0, 0.005, x=0.0, 0.005, 0.01, 0.03, and 0.05) nanoparticles, (**b**) enlarged view of pristine and co-doped ZnO (101) peak, and (**c**) plot showing increment in crystallite size(**D**) versus Cu/Al co-doped ZnO.

The X-ray diffraction approach was used to investigate structure of materials, determine the crystallinity of the samples, nanoparticle size, phase purity, and lattice parameters. The polycrystalline peaks observed in Fig. 1a match to the Miller Indices (100), (002), (101), (102), (110), (103), (200), (112), and (201) planes of pure wurtzite hexagonal structure. Among these peaks, the (101) plane (2$\theta$ = 36.3$^\circ$) exhibits the highest intensity peak, indicating a highly crystalline and well-defined nature. The absence of impurity peaks in all samples (pH $\sim$ 9-10) confirms the complete decomposition of all precursor solutions, which matches with previous reports^34,35^. The peaks corresponding to all the samples closely match with the JCPDS data (01-089-0510,01-079-0206). Analysis of the (101) plane peak positions for pristine ZnO and Al(3) reveals a shift to higher 2$\theta$ values compared to other co-doped samples (Fig. 1b). This right peak shift confirms the substitution of copper and aluminum ions into the ZnO matrix^36^. The shifting of peaks occurred due to smaller ionic radii of Al$^{3+}$ and Cu$^{2+}$ comparing to Zn$^{2+}$ ion^37^.

The line broadening observed in the main (101) peak of pristine and co-doped ZnO materials indicates the nanometer range of the synthesized samples. The crystallite size (*D*) of the prepared powder samples were determined utilizing the Scherrer equation, which uses line broadening of the (101) peak^38^. The Scherrer formula is given by: $D= 0.9\lambda / \beta \cos \theta$, where *D* depicts crystallite size, $\lambda$ is referred as the wavelength of the X-ray (1.5404 Å), $\beta$ represents full width at half maximum (FWHM) of XRD peak, and $\theta$ denotes angle of diffraction peak.

The samples prepared by sol-gel method exhibit a range of crystallite size between 43 and 90 nm, as summarized in Table T1 (supplementary text). The results reveal that the incorporation of copper and aluminum dopants (Cu(0.5) and Al(0.5)) leads to increment in crystallite size when compared with pristine sample. In particular, crystallite size of Cu(0.5) is larger when compared to Al(0.5). Furthermore, both Cu(0.5) and Al(3) exhibit larger crystallite sizes compared to the pristine and other co-doped samples, as observed in Fig. 1c.

**Figure 2:**
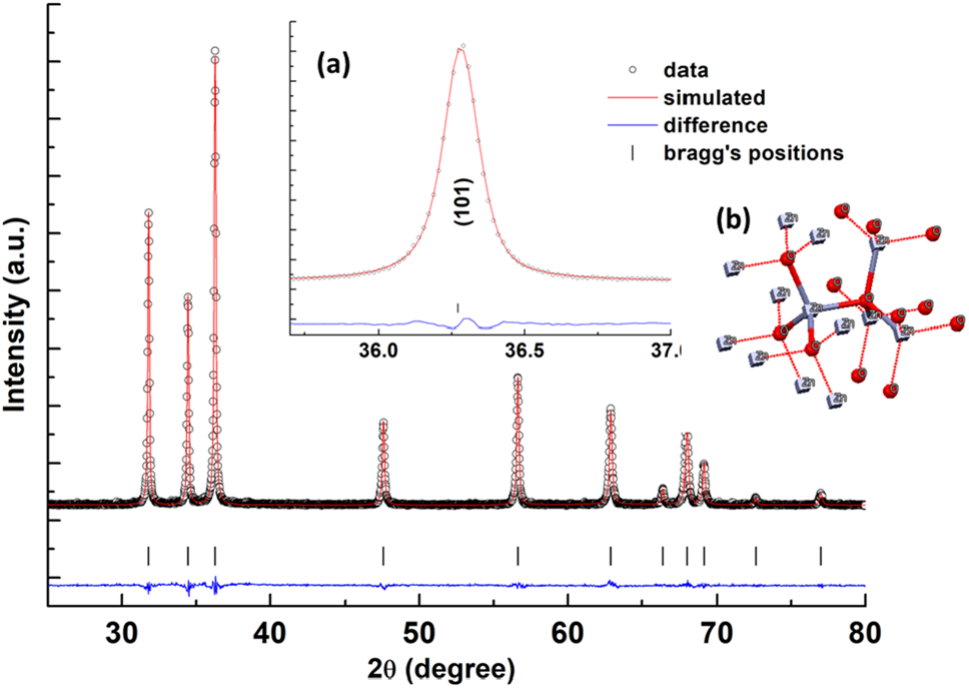
Rietveld refinement of pristine ZnO(*P*63*mc)*. The XRD data is represented by open circles, while the lines depict theoretical fits to the observed X-ray data. The vertical bars indicate the Bragg reflections corresponding to the planes. The difference pattern between observed data and theoretical fit is presented at bottom. The inset features: (**a**) an enlarged view of the pristine ZnO(101) peak, and (**b**) the refined parameters utilized for the determination of electronic structure of pristine sample of ZnO.

The crystal structure investigation has performed utilizing Rietveld refinement of pristine and co-doped samples of ZnO XRD data^39^. The Rietveld refined XRD patterns by considering the *P*63*mc* space group for the pristine ZnO hexagonal structure, are revealed in Fig. 2, confirming presence of ZnO wurtzite structure. The other Rietveld refineed patterns of co-doped samples compared with pristine ZnO is shown in Fig. S1 (supplementary text). The calculated structural parameters of synthesized samples are recorded in Table T1 (supplementary text), demonstrating fitting quality of the experimental data. The non-variant *c*/*a* ratio (1.602) is less than the ideal value of 1.633, indicating an increasing behavior of the *u*-parameter (bond length parallel to c-axis), while keeping the tetrahedral distance uniform through deformation of tetrahedral angles, considering long-range interactions^40,41^. With the modification of the *c*/*a* ratio, wurtzite structure changes from ideal arrangement in real zinc oxide lattice. This variation may be because of lattice instability and ionicity. The presence of point dislocations, like oxygen vacancies, extended disorders, and zinc antisites, increases the lattice constant in crystal lattice of ZnO. The small variations in lattice parameters are associated with the small differences in electronegativity and ionic radius between Zn$^{2+}$ (0.74 $\text{\AA }$) and Al$^{3+}$ (0.54 $\text{\AA }$) atoms^42^.

**Table 1:** Rietveld refined parameters of pristine and various co-doped samples of ZnO.

Sample name	Pr	Cu(0.5)	CuAl(0.5)	Al(1)	Al(3)	Al(5)
a=b (Å)	3.2481	3.2481	3.2476	3.2477	3.2459	3.247
c(Å)	5.2029	5.2029	5.2030	5.2027	5.1993	5.2026
*c*/*a*	1.602	1.602	1.602	1.602	1.602	1.602
Bragg R-factor (%)	1.79	1.69	1.50	1.65	1.62	1.61
RF-factor (%)	1.45	1.13	1.15	1.16	1.14	1.12
No. of reflections	10	13	10	11	11	11
$\alpha (^\circ )$	90	90	90	90	90	90
$\beta (^\circ )$	90	90	90	90	90	90
$\gamma (^\circ )$	120	120	120	120	120	120
Volume (Å$^3$)	48.049	47.536	47.524	47.512	47.439	46.84
R$_{WP}$ (%)	59.20	58.536	59.524	58.325	59.356	58.951
Bond length (Å)	1.9722	1.991	1.9777	1.9769	1.9765	1.9761
Bond angle ($^\circ$)	108.0	110.9	108.0	110.9	110.8	110.5

### Strain, stress and energy density analysis

To analyze stress, strain, and energy density, originating due to the crystal structure energy (Gibbs free energy), Williamson and Hall (W-H) plots were investigated using the variation in the plane parameters as per the equations (Eq. 1–Eq. 3). W-H utilized convolution to explore the impact of size and strain parameters, resulting in broadening of peaks. The broadening of the peak caused by crystal size $\beta$ (determined by the Scherrer formula) is dependent on the angle, following a variation of $1/\cos \theta$, while the broadening due to strain (represented as $\beta _e = 4\varepsilon \tan \theta$) varies as $\tan \theta$. Therefore, the W-H equation is:

1 $$\begin{aligned} \beta _{hkl}\cos \theta = \frac{k\lambda }{D} + 4\varepsilon \sin \theta \end{aligned}$$

Equation (1) is referred to as Uniform Deformation Model (UDM), assuming the value of strain within the material is uniform in every direction, considering crystal to be isotropic. Similarly, the Uniform Stress Deformation Model (USDM) is construct on certain assumptions. Hence, Hooke’s relation, which asserts a linear proportionality between strain and stress ($\sigma = Y\varepsilon$, here *Y* is Young’s modulus) is employed for the calculation of stress. Consequently, the W-H equation (Eq.  1) is refined as follows:

2 $$\begin{aligned} \beta _{hkl}\cos \theta = \frac{k\lambda }{D} + \frac{4\sigma \sin \theta }{Y_{hkl}} \end{aligned}$$

The Young’s modulus determined using the XRD analysis  for ZnO nanoparticles (hexagonal) was 119 GPa, a similar value is also reported by Zak et al.^43^. The Uniform Deformation Energy Density Model (UDEDM) is employed for the estimation of crystal energy density. However, the assumption of homogeneous isotropic nature of crystal is not valid within the Uniform Deformation Model (UDM). Therefore, when considering the energy density within elastic limits, the expression of Hooke’s law is: $u = (\varepsilon ^2Y_{hkl})/2$ The W-H equation (Eq.  2) can be rewritten in the form of Eq. (3):

3 $$\begin{aligned} \beta _{hkl}\cos \theta = \frac{k\lambda }{D} + 4\sin \theta \left( \frac{2u}{Y_{hkl}}\right) ^{1/2} \end{aligned}$$

Linear regression is performed to get W-H plots, as depicted in Figs. S11 to S16 (supplementary text), where the slope yielded insights into energy density, stress, and strain, while intercept is utilized for the estimation of crystal size^39^. Within aforementioned models, lattice strain, lattice stress, and energy density are calculated with certain approximations. Remarkably, the crystallite size acquired utilizing these models exhibited a strong agreement with determined average size through Rietveld refinement.

### Field-emission scanning electron microscopy (FE-SEM) analysis

**Figure 3:**
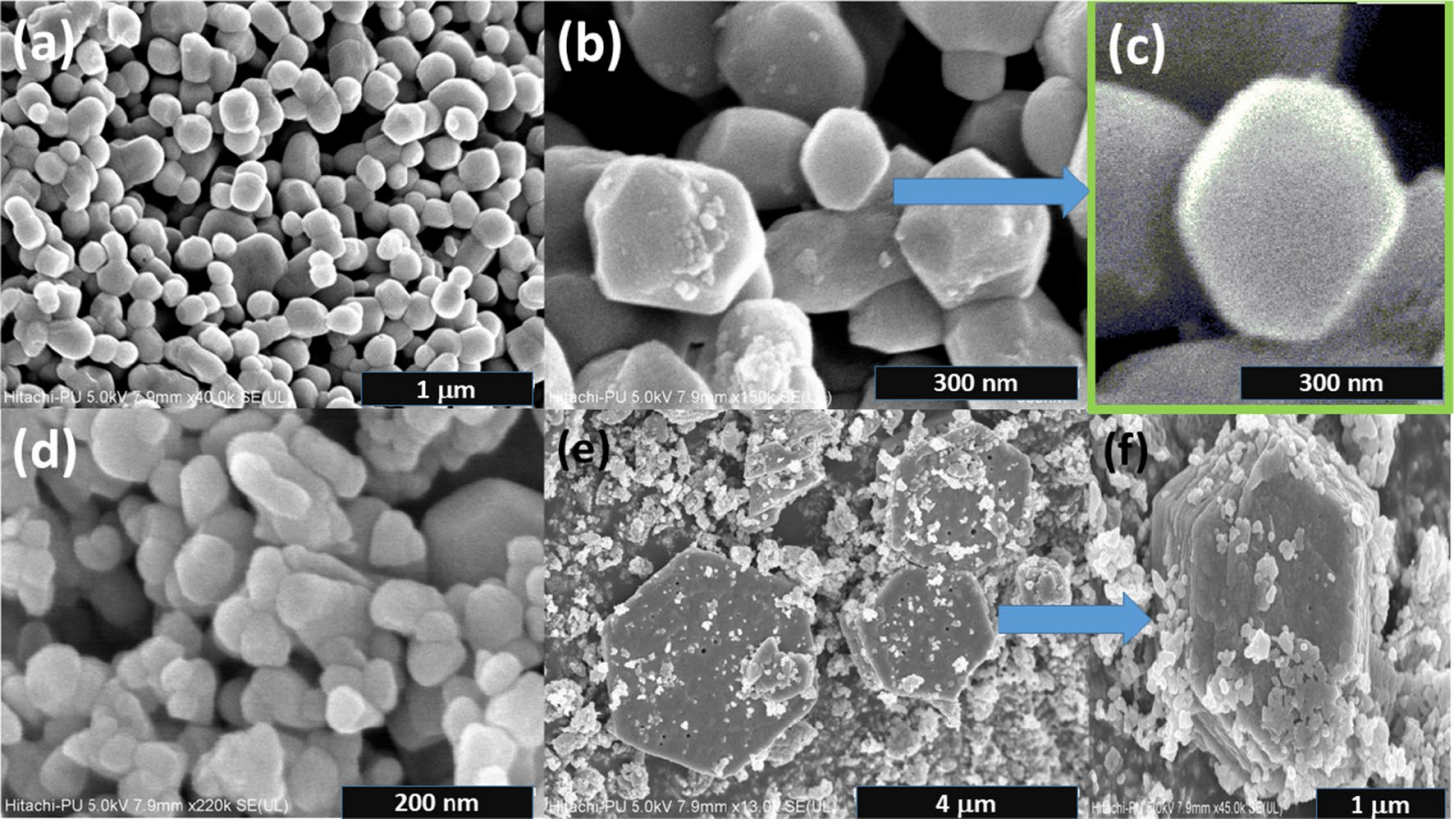
FE-SEM micrographs of (**a**) Cu(0.5) nanoparticles, (**b**) pseudo-hexagonal-shaped Cu(0.5) nanostructure, (**c**) magnified image of the pseudo-hexagonal-shaped Cu(0.5) nanostructure, (**d**) Al(0.5) nanoparticles, (**e**) pseudo-hexagonal-shaped Al(0.5) nanostructure, and (**f**) magnified image of the pseudo-hexagonal-shaped Al(0.5) nanostructure showing nanoburger.

**Figure 4:**
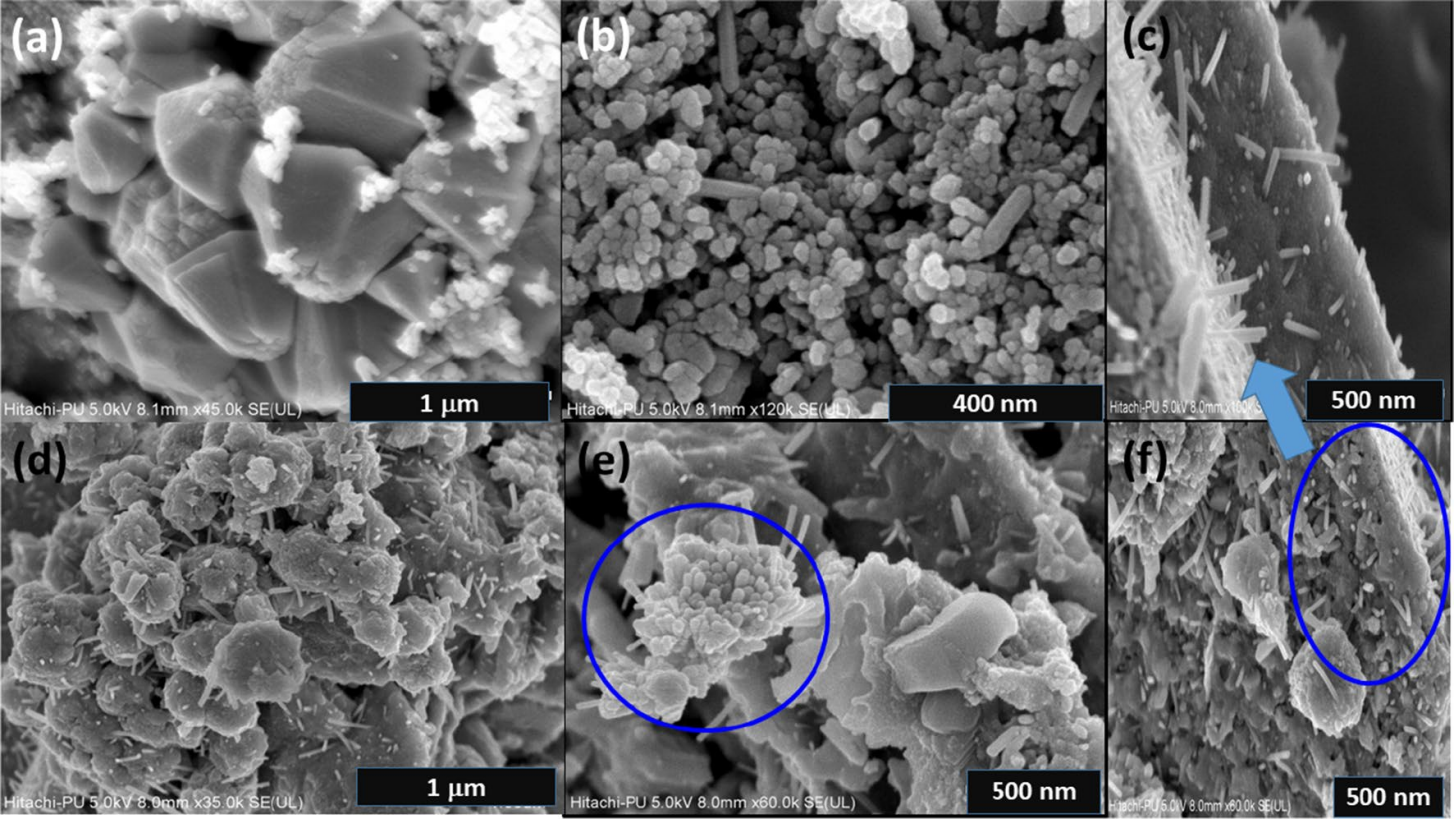
FE-SEM micrographs of (**a**) Al(1) pyramid-like nanostructures, (**b**) Al(1) nanorods, (**c**) magnified image of Al(5) nanorod, (**d**) Al(5) cauliflower-like structures with nanorods, (**e**) Al(5) cauliflower-like structure, and (**f**) Al(5) nanorods.

**Figure 5:**
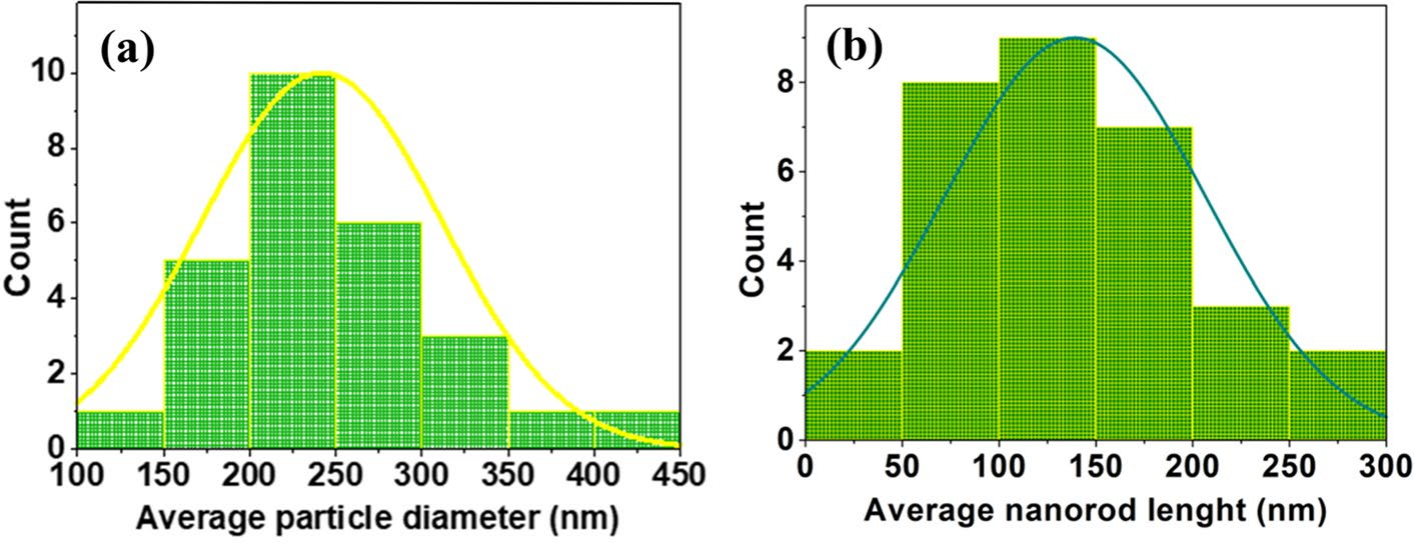
Histogram for (**a**) average diameter of Cu(0.5) nanoparticles, and (**b**) average length of Al(5) nanorods.

Field emission scanning electron microscopy (FE-SEM) micrographs (Figs. 3 and 4) reveal the diverse morphologies of the ZnO nanostructures, including pseudo-hexagonal structures, nanorods, pyramids, and flowers. The pseudo-hexagonal structures exhibit grain size of 238.2 nm, while average length of nanorods is 157.2 nm. Figure 5a,b shows the histogram for average diameter of Cu(0.5) nanoparticles, and average length of Al(5) nanorods, respectively. The nanostructures exhibit high density and uniformity, likely attributed to the growth mechanism.

Figure 3a,b depict the formation of well-distributed pseudo-hexagonal-shaped copper-doped ZnO nanostructures (Cu(0.5)), consistent with the previous observations^39^. A magnified view of the pseudo-hexagonal-shaped (Cu(0.5)) nanostructure is shown in Fig. 3c. The substitution of aluminum doping leads to nanoparticle agglomeration, as evident in Fig. 3d,e. The agglomeration of pseudo-hexagonal nanoburgers (enlarged view of Fig. 3e) is observed in Fig. 3f.

The FE-SEM images in Fig. 4a demonstrate the formation of well-dispersed pyramid-like nanostructures (Al(1)). These nanopyramid-like assemblies consist of dozens of pseudo-hexagonal rod-like nanostructures, acting as building block units (Fig. 4b). With increased aluminum doping concentration, uniformly distributed micro-flowers composed of nanorods (Al(5)) are formed, as depicted in Fig. 4d. The cauliflower shape of Al-doped ZnO nanostructure is highlighted by the marked circle in Fig. 4e. Similar FE-SEM images of nanoflowers and nanorods are described in previous reports^44,45^. The flower-like ZnO nanostructures appear to form due to the branching of central rods rather than the aggregation of rod-like nanostructures. The magnified image of the nanorod marked in the circle of Fig. 4f can be seen in Fig. 4c^46^. Such hierarchical flower-like nanostructures possess numerous pores/radicals, which can enhance the sensing properties of photodetectors. Therefore, the FE-SEM images demonstrate the diverse morphologies of ZnO nanostructures, which can be attributed to different doping concentrations. The flower-like nanostructures, with their abundance of pores, have the potential to improve the sensing properties of photodetectors.

The typical process of forming ZnO nanostructure can be explained by following reactions^39,47^:

4 $${\text{Zn}}({\text{CH}}_{3} {\text{COO}})_{2} \cdot 2{\text{H}}_{2} {\text{O}} + 2{\text{NaOH}} + 2{\text{H}}_{2} {\text{O}} \to {\text{ Zn(OH}})_{2} \downarrow + ~2{\text{CH}}_{3} {\text{COONa}} + 4{\text{H}}_{2} {\text{O }}$$

5 $${\text{Zn}}({\text{OH}})_{2} + 2{\text{OH}}^{ - } \to {\text{ }}[{\text{Zn}}({\text{OH}})_{4} ]^{{2 - }}$$

6 $$({\text{Zn}}({\text{OH}})_{4} )^{{2 - }} \leftrightarrow {\text{ZnO}} \downarrow + ~{\text{H}}_{2} {\text{O}} + 2{\text{OH}}^{ - } .$$

These reactions describe the transformation of Zn(CH$_3$COO)$_2\cdot$2H$_2$O into Zn(OH)$_2$ through the addition of NaOH and subsequent generation of [Zn(OH)$_4$]$^{2-}$. Finally, [Zn(OH)$_4$]$^{2-}$ endure a reaction to prepare ZnO nanoparticles along with release of water and OH$^-$ ions. These reactions perform a crucial part in synthesis of ZnO nanostructures.

**Figure 6:**
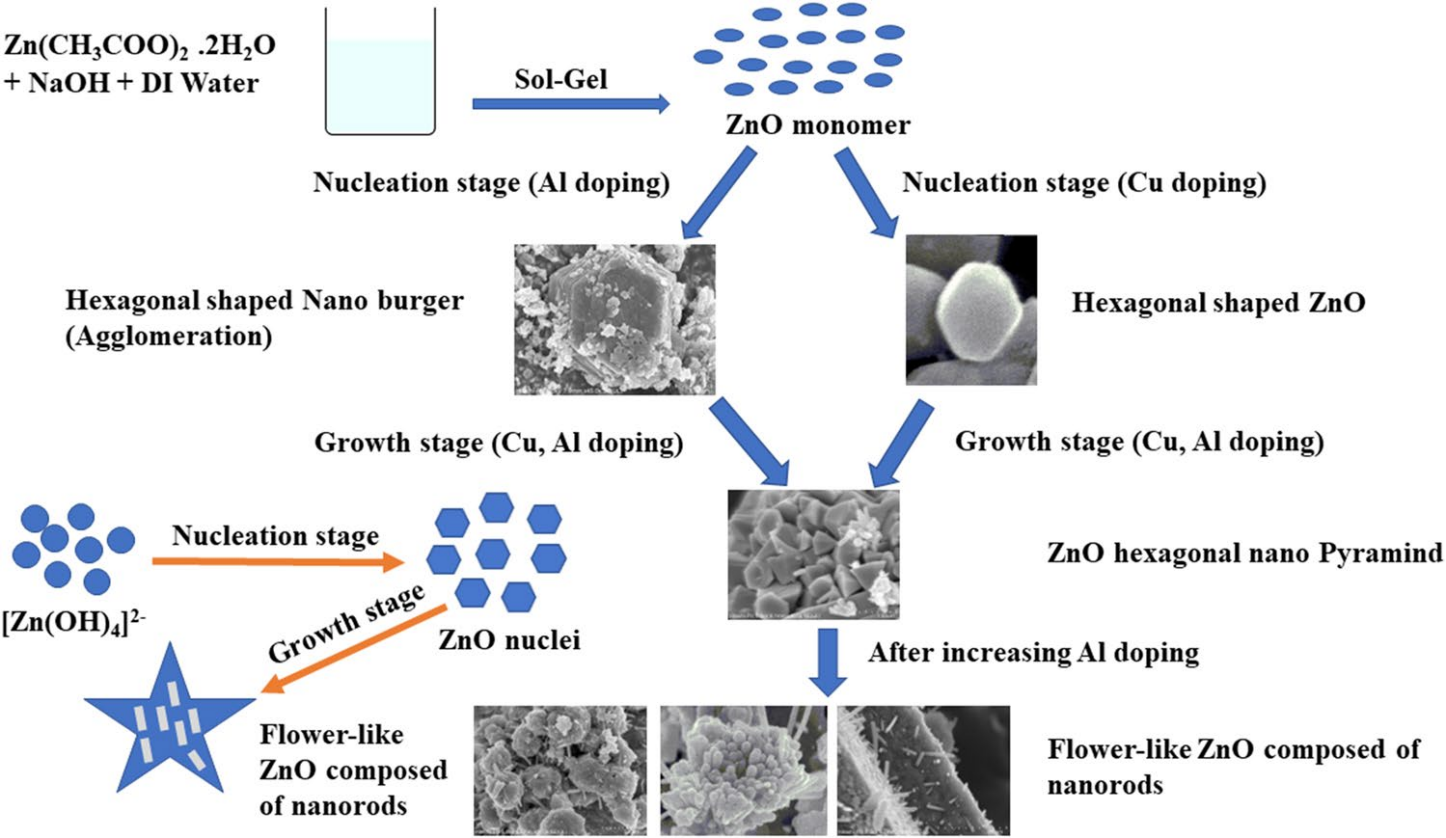
Nucleation and growth mechanism for flower like ZnO wurtzite nanostructure.

The growth mechanism of nanostructures of ZnO can be understood by considering the nucleation and crystal growth processes. It is hypothesized that the growth mechanism is governed by a nucleation/crystal growth approach, wherein nucleation process arise initially, following crystal growth process. The nucleation process is relatively slow compared to the faster crystal growth process, especially at higher pH values. During the nucleation process, the [Zn(OH)$_4$]$^{2-}$ growth component readily attaches to surface of ZnO seed, inducing the growth of seed nuclei along the *c*-axis and the generation of cauliflower-shaped nanostructures. With the increase of the reaction time and annealing temperature, re-nucleation of side branches occurs, resulting in the growth of nanorods. The mechanism underlying nucleation and growth processes involved in this preparation can be elucidated through Fig. 6, which provides further insights into morphological modification of the ZnO nanostructures.

The elemental percentage calculated from Energy Dispersive X-ray (EDX) microanalysis as shown from Figs. S3, S4, S5, and S6 (supplementary text) are tabulated in the isnets of these figures.

### Diffuse reflectance spectroscopy (DRS) analysis

**Figure 7:**
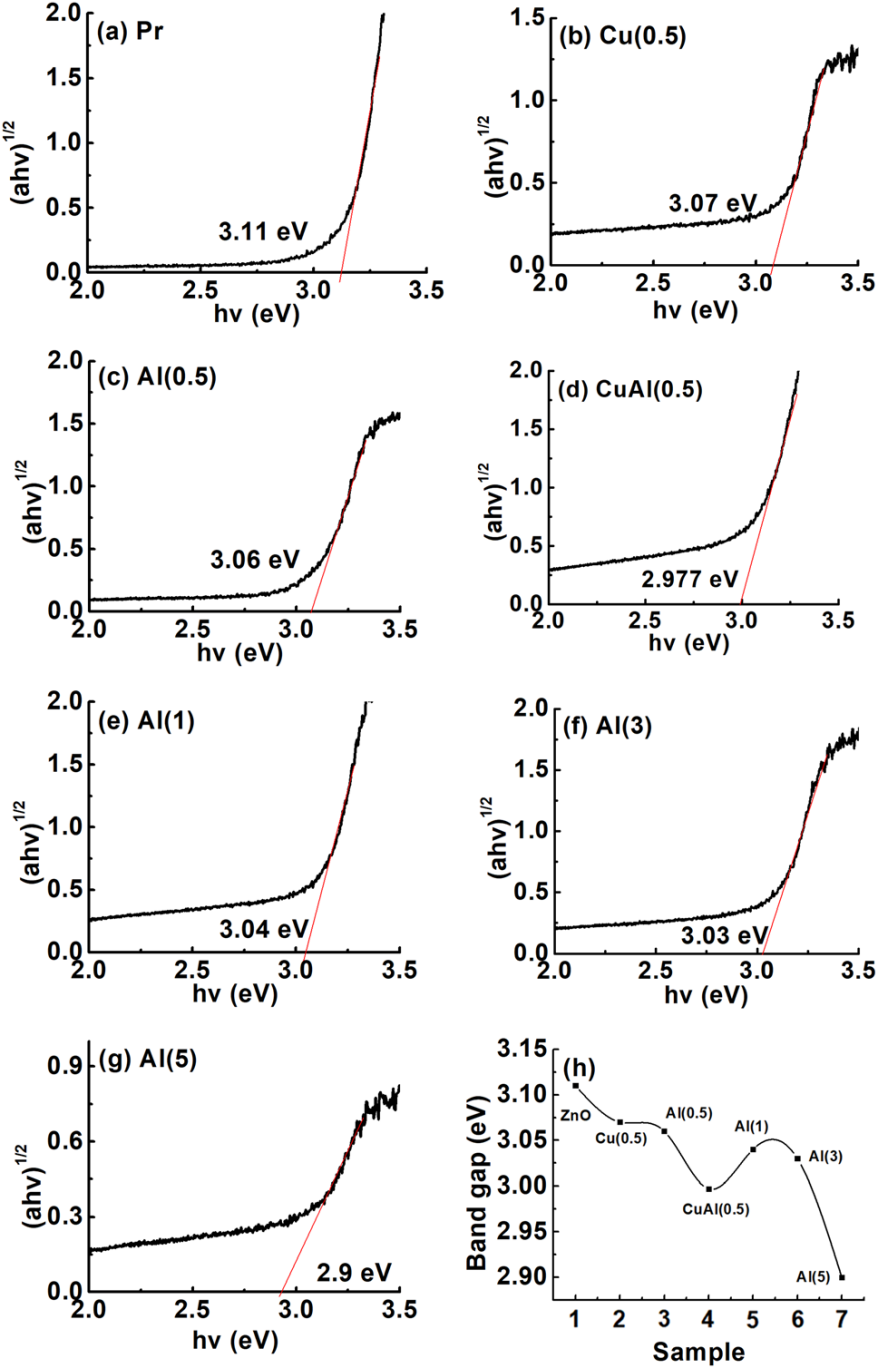
Band gap calculated by Tauc’s plot method from diffuse reflective spectroscopic data for (**a**) Pr(ZnO), (**b**) Cu(0.5), (**c**) Al(0.5), (**d**) CuAl(0.5) (**e**) Al(1), (**f**) Al(3), (**g**) Al(5), and (**h**).

The absorption spectrum of pristine and various co-doped zinc oxide were recorded in range of 200–800 nm. The absorption edges of all the pristine and co-doped samples were found to be similar, indicating a small difference in energy bandgap (Fig. 7).

Diffuse Reflectance Spectroscopy (DRS) techniques are utilized for the study of band gap. The band gap energy in semiconductors represents energy needed to excite an electron toward the conduction band. The band gap energy of pristine and co-doped zinc oxide is calculated utilizing the Tauc, Davis, and Mott relation^48^ for indirect band-gap:

7 $$\begin{aligned} (\alpha h \nu )^{1/2} = A( h \nu - E_g) \end{aligned}$$

Here, $\alpha$, $\nu$, $E_g$, and *A* represent absorption coefficient, frequency of light, band gap energy, and proportionality constant, respectively.

The band gaps are evaluated by plotting ($\alpha$h$\nu$)$^{1/2}$ against Energy (E) (Fig. 7). The band gap values are calculated by extrapolating straight line to the intercept axis (Fig. 7)a,b,c,d,e,f and g. The calculated band gap values ranged from 3.11 to 2.9 eV, consistent with previous reported values of 3.1–3.2 eV by Mott et al.^49^.

In the present study, band gap of co-doped samples reduced due to variations in dopant size, host material, and annealing effects, leading to defect formation. The absorption edge shifted toward longer side of wavelength, indicating the formation of extra energy levels within band gap of zinc oxide with the incorporation of Al dopants into Cu-ZnO matrix. Therefore, less energy is needed for the excitation of electrons into the conduction band, resulting in increased charge transfer. The decrease in the band gap of zinc oxide with increment in Al dopant concentration can be attributed to increase in grain size and decrease in carrier concentration^50^ (Fig. 7h), consistent with the analysis of XRD showing an increase in crystallite size.

### Analysis of photoluminescence spectra (PL)

**Figure 8:**
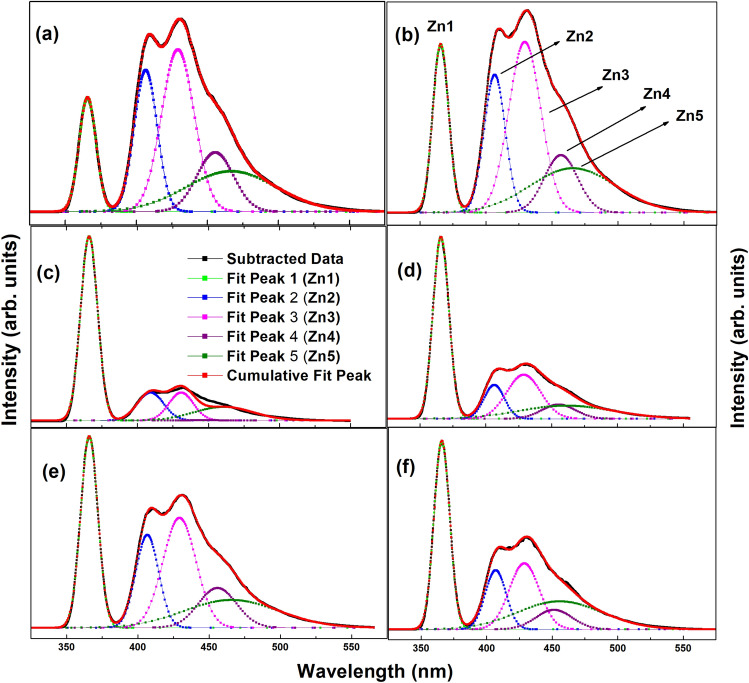
Photoluminescence spectra of (**a**) Pr(ZnO), (**b**) Cu(0.5), (**c**) Al(0.5), (**d**) Al(1), (**e**) Al(3), and (**f**) Al(5).

The pristine ZnO sample exhibits a large intensity ratio of visible peak/ultraviolet, indicating fluorescence. Photoluminescence (PL) analysis was conducted to investigate optical properties of co-doped ZnO nanostructures (Fig. S10 in supplementary text). The peak analysis of PL for pristine and co-doped samples is presented in Fig. 8. The different peak positions (Zn1, Zn2, Zn3, Zn4, Zn5) and their corresponding values of full-width at half-maximum (FWHM) are shown in Table T2 (supplementary text). The Zn1 peak at 366 nm in all samples depicts recombination of free charge carrier. The wavelength values of Zn2 peaks for all samples exhibit an increasing trend compared to the corresponding Zn1 peaks. Similarly, the wavelength values of all other peaks (Zn3, Zn4, Zn5) increase in comparison to the Zn1 peaks. In the case of Al(5) samples, all peaks show a decrease in wavelength compared to pristine ZnO, indicating reduction in recombination rate of free excitons. The decrease in intensity of the second peak (Zn2) with varying doping amounts may be because of the net increment in defects, like zinc or oxygen vacancies^51,52^, which can be correlated with the O K-edge NEXAFS analysis revealing existence of oxygen vacancies, discussed in next sections.

The photoluminescence spectra of ZnO and (Al,Cu) co-doped zinc oxide show the presence of two peaks. The strong emission peak nearby 366 nm (ultraviolet region) is ascribed to increased number of charge carrier recombination rates in the near-band edge (NBE). A lower number of free electrons in the system leads to a decrease in conductivity and, consequently, a decrease in photosensing in photodetectors. The broad emission peak observed in green band is ascribed to the creation of oxygen vacancies^53,54^. Photoluminescence (PL) serves as an optical approach to identify defects in a sample. The UV peak arises from the fast recombination of charge carriers. The decreased band gap observed in co-doped ZnO nanostructure, compared to pristine zinc oxide (3.37 eV), suggesting the presence of significant defect levels in prepared zinc oxide sample. The narrowing in the band gap is ascribed to donor impurities creating energy levels nearby conduction band. Therefore, when the PL intensity is weak, the recombination rate of charge carriers (photo-generated) slows down, leading to an increase in conductivity and enhanced photosensing in photodetectors^53,54^. The PL intensity peaks of Al-doped and (Cu,Al) co-doped zinc oxide (Fig. 8c,d,e and f) are lower compared to PL spectra of ZnO and Cu-doped ZnO (Fig. 8a,b), indicating an improvement in the optical properties of co-doped zinc oxide. This can be ascribed to the presence of doped ions creating numerous electron traps, suppressing the charge carrier recombination rate and promoting charge transfer in the ZnO system. Comparing all the PL intensities, it is evident that the co-doped zinc oxide samples (Fig. 8c,d,e and f) exhibit lower recombination rates and thus exhibit better optical properties^51,52^.

In the zinc oxide lattice, the structure is hexagonal close-packed (HCP) with Zn$^{2+}$ ions surrounded by six O$^{2-}$ ions. The introduction of aluminium and copper ions into the zinc oxide lattice can modify the hybridization state of the surrounding atoms and enhance optical and electronic properties of the material ZnO^55^. The hybridization state of aluminium and copper ions depends on their coordination environment and the specific doping conditions. The hybridization state of copper ions can range from $sp^2$ to $sp^3d^2$ depending on their coordination with two, three, or six oxygen atoms. Similarly, the hybridization state of aluminium ions can range from $sp^3$ to $sp^3d^2$ depending on their coordination environment. Determining the exact hybridization state of aluminium and copper ions in a specific Al and Cu co-doped ZnO structure requires detailed analysis of the crystal structure and bonding environment using advanced characterization techniques such as electron microscopy, electron spectroscopy, and XRD.

However, in general, we can discuss the hybridization of aluminium and copper atoms in the ZnO structure. In Cu-doped ZnO, copper replaces some of the zinc atoms in the lattice. The partially filled *d*-orbitals of copper hybridize with Zn and O atoms, resulting in a combination of *s* and *d* orbitals, known as *sp*-*d* hybridization. In Al-doped ZnO, aluminium replaces some of the zinc atoms in the lattice. The partially filled *p*-orbitals of aluminium hybridize with Zn and O atoms, resulting in a combination of *s* and *p* orbitals, known as *sp*-*p* hybridization. The overall hybridization of aluminium and copper co-doped ZnO can be complex and depends on the specific arrangement of atoms and their bonding nature (Fig. S8 in supplementary text)^56,57^.

### FT-IR analysis

Fourier transform infrared spectroscopy (FT-IR) is a valuable approach utilized for the identification of various functional groups in compounds. In the range of 400–4000 cm$^{-1}$, Fig. S2 displays FT-IR spectra of nanosized Al$_x$Cu$_{y}$Zn$_{1-x-y}$O powders (with *y* values of 0.0, 0.005, and *x* values of 0.0, 0.005, 0.01, 0.03, and 0.05). The spectra exhibit various bonds, including Zn–O, O–H, Al–O, Cu–O, C–O, COO$^-$, and CO$_2$. The presence of C–O and O–H bands may be attributed to sol-gel technique and organic residues. Around 2300 cm$^{-1}$, the absorption band is correlated with atmospheric air. The generation of Cu–O and Al–O bonds confirms the successful incorporation of aluminium and copper atoms into the ZnO lattice. Notably, annealing at 400 $^\circ$C leads to a significant increase in transmission around the Al–O bond frequency, indicating the effectiveness of this annealing temperature for the successful substitution of aluminium atoms. The absorption peaks observed at 1600 and 3450 cm$^{-1}$ correspond to stretching vibrations of O–H bonds, ascribed to water absorbed by the sample surface. The absorption peak at 2380 cm$^{-1}$ suggests presence of CO$_2$ molecules in environment^58^. Additionally, Figure S2 (supplementary text) demonstrates that in pristine sample, a strong absorption peak observed at 435 cm$^{-1}$ corresponds to stretching vibrations of the Zn-O bond. In the co-doped material, additional absorption band nearby 620 cm$^{-1}$ is noticed, associated with vibration of Cu–O bond^59^.

### Near edge X-ray absorption fine structure(NEXAFS) analysis

Near-edge X-ray absorption fine structure (NEXAFS) is a aggressive approach employed to explore various aspects of electronic structure, such as hybridization, crystal field strength, chemical valency of cations, and assess the presence of sample vacancies. This technique involves the excitation of core electrons through the absorption of photon energy, with the transitions governed by dipole selection rules. It provides valuable insights into the electronic properties and local chemical environment of the material under investigation^60^.

#### O K-edge NEXAFS

**Figure 9:**
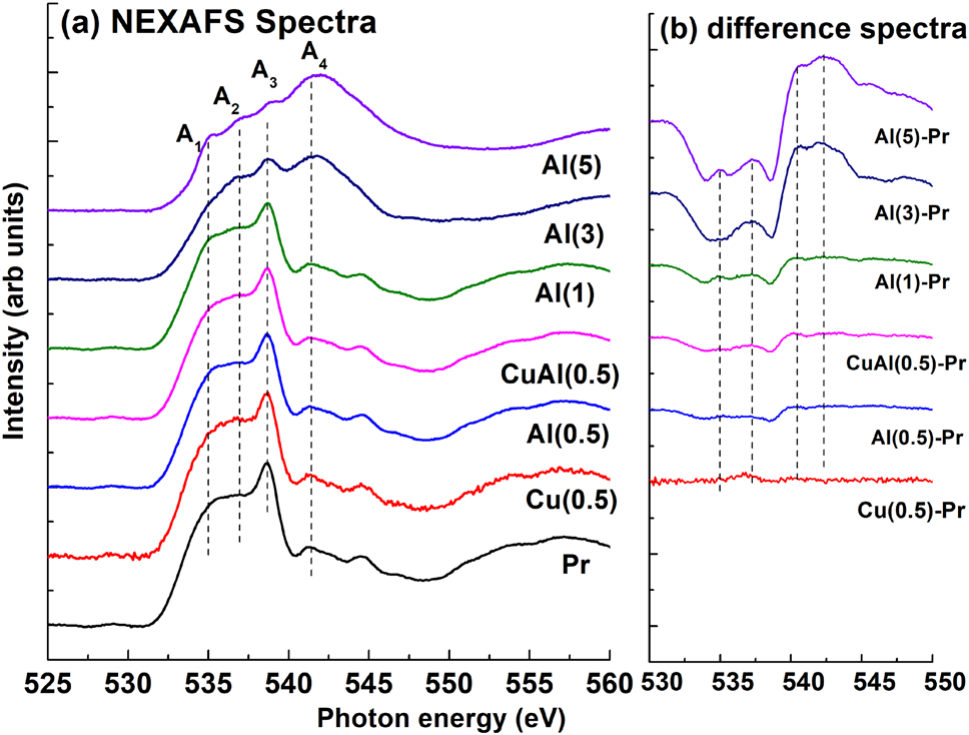
Normalized O K-edge NEXAFS spectra for (**a**) Al$_x$Cu$_{y}$Zn$_{1-x-y}$O (y=0.0, 0.005, x=0.0, 0.005, 0.01, 0.03, and 0.05), and (**b**) difference spectra showing the variation in spectral features with Al-Cu co-doping.

To understand co-doping effect, NEXAFS spectra of Al$_x$Cu$_{y}$Zn$_{1-x-y}$O (*y*=0.00, 0.005, *x*=0.00, 0.005, 0.01, 0.03, and 0.05) at O K-edge are described from Fig. 9. The O K-edge represents the 1*s* core state transition to empty derived states of oxygen (2*p*), hybridized with small 3*d* and wide 4*sp* bands^61–63^. From Fig. 9a, O K-edge spectra (normalized) of Cu(0.5), Al(0.5), CuAl(0.5), and Al(1) resemble that of pristine ZnO. However, the spectra of Al(3) and Al(5) are different from pristine ZnO, suggesting a modified nanostructure. These spectra exhibit a normalized behavior with the same area in the range of 550 and 570 eV. In Fig. 9a, four peaks (A$_1$, A$_2$, A$_3$, and A$_4$) are recognized in this spectra, centered at 535, 537, 539, and 542 eV, respectively. The distinctive spectral feature between the energy range of 535–538 eV (denoted by A$_1$ and A$_2$) can be ascribed to hybridization of O 2*p* state with Zn, Cu, Al 3*d*/4*s* states, the region in between 538 and 542 eV (A$_3$ and A$_4$) corresponds to hybridization of O 2*p* state with Zn, Cu, Al 3*p* states.

By comparing the spectrum of Al(3) and Al(5) with pristine ZnO and other doped samples, two additional spectral features at 538 eV (A$_3$) and 542 eV (A$_4$) are observed. The intensity of these features decreases and increases with Al doping, respectively, indicating a modification of the local electronic structure of these Al doped Cu-ZnO nanostructures^64^. Therefore, this new feature at 538 eV, nearby the minimum of conduction band, may be assigned to result of dopant 3*d* hybridized with O 2*p* state. The broadening of spectral features observed in higher Al co-doped zinc oxide at 538 eV (A$_3$) and 542 eV (marked by A$_4$) ascribed to the appearance of oxygen vacancies or morphology effect observed in FESEM micrographs. The presence of new spectral features and increment in broadening imply that Al atoms fill up interstitial sites^65^ in the zinc oxide surrounding oxygen vacancies. Consequently, because of the presence of oxygen vacancy, more charge can be transferred from the doped Al ions, resulting in Al ions tetrahedrally coordinated to oxygen sites in the ZnO matrix^66^. The broadening of these spectral features in Al(5) is higher compared to the other samples, indicating high assembly of oxygen-related defects and magnetic moment, ascribed to the appearance of oxygen vacancy and morphological effect^67,68^. Figure 9b shows the difference spectra illustrating the variation with Al–Cu co-doping.

Chen et al.^64,69^ provide an explanation for the spectral variation observed, which ascribed with the concept of charge transfer. As depicted from Fig. 9, intensity of highly Al co-doped ZnO is observed to decrease compared to pristine ZnO. This reduction can be attributed to a higher occupation degree of the O 2*p* state in zinc oxide, which is explained from the concept of enhanced charge transfer from Al (adsorbed) to O^64^. The presence of multiple electron traps and oxygen vacancies can be correlated with photoluminescence (PL) and Zn L-edge NEXAFS spectra. The hybridization between the orbitals of 3*p* incorporated Al and orbitals of 2*p* adjacent to O promotes charge transfer from Al to O^64^, which is also explained in Fig. 12. This change in spectral shape of doped ZnO indicates the occurrence of significant morphological changes in the material.

It has suggested that Al can either be substituted into the lattice of ZnO system by incorporating for Zn ions or create a layer onto the grain boundaries or surface of ZnO powder^64^. In the XRD pattern, no generation of secondary phase is observed; however, possibility of Al$_2$O$_3$ phase cannot be ignored^70^. In the case of pristine ZnO, the A$_3$ peak is clearly visible, but in heavily Al-doped ZnO, the A$_3$ peak is smoothed, indicating the existence of structural disorder as correlated with the FESEM micrographs (Figs. 3 and 4 ) of co-doped ZnO. The effects of charge transfer with the incorporation of O by Al and Cu are reflected in XAS intensity due to the different backscattering properties.

#### Zn L-edge NEXAFS

**Figure 10:**
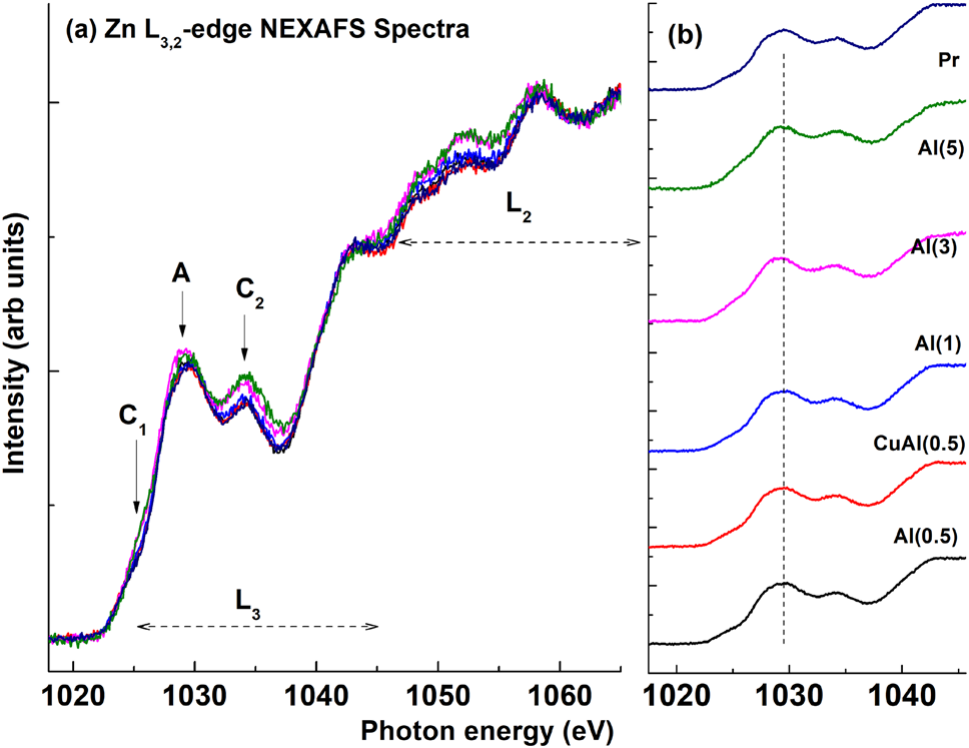
Normalized Zn L-edge NEXAFS spectra for (**a**) Al$_x$Cu$_{y}$Zn$_{1-x-y}$O (*y*=0.0, 0.005, *x*=0.0, 0.005, 0.01, 0.03, and 0.05), and (**b**) difference spectra showing the variation with Al-Cu co-doping.

NEXAFS measurements are conducted to examine the Zn L-edge, providing knowledge of the unoccupied Zn *d* and Zn *s* -states. Figure 10 illustrates the normalized Zn L$_{3,2}$-edge of Cu-incorporated zinc oxide. The L$_3$ and L$_2$ regions correspond to the Zn (2*p*) to Zn (4*s*) and Zn (3*d*) antibonding state, respectively, following Mott selection rules^71^. The spectral features in the L$_3$ region represent electron transition from Zn (2*p*) to Zn (3*d*) state.

The pre-edge feature, indicated by a downward arrow at C1, is influenced by the Zn 4*s* band transition. In contrast, the spectral feature in the L_2_ region results from multiple overlapping bands^72^, exhibiting symmetrical position and shape. This also suggests, Zn defect should not contribute to magnetic properties of copper-incorporated zinc oxide. The Zn L-edge spectrum in ZnO generally arises from core shell electrons. The degenerate states 2*p*_3/2_ and 2$p_{1/2}$ emerge due to spin-orbit coupling, leading to multiplets centered at 1026 and 1034 eV. The crystal field (octahedral) raise degeneracy of 2$p_{3/2}$ and 2$p_{1/2}$ levels, resulting in the creation of t$_{2g}$ and e$_g$ sub-band symmetries^73^. The fine structure multiplet arises from two effects: (1) interaction in-between the 3*d* (electron) and 2*p* core hole, and (2) crystal field created by neighboring ions at a Zn$^{2+}$ site^74^.

When investigating Al co-doping in the Cu–ZnO system, it is observed that up to 1 at.% of Al does not alter the degenerate levels. However, for Al doping above 1 at.%, the splitting of levels becomes evident, as indicated by C_1_ (t$_{2g}$)-C$_2$ (e$_g$). The crystal field splitting results in sharp peaks due to the separation of degenerate levels. The peak of the Al(5) sample is shifted in energy due to an increasing nature of the crystal field parameter 10*Dq* (marked by C$_2$), which is consistent with the results from XRD, PL, FESEM, and O K-edge analysis (Fig. S9 in supplementary text).

To determine 10*Dq* in Fig. 10, we examine the sharp pre-edge feature that quickly varies with the change in 10*Dq*. The shifted peak of the Al(5) sample is also observed in the pre-edge feature, marked by C$_1$, while another feature marked by C$_2$ is also present. Additionally, there is a shifted peak of Al(3) marked as ‘A’. These shifted peaks can be sensitive to morphology and can be correlated with FESEM micrographs. Therefore, by considering all the results, it appears that heavy doping of Al in Cu-ZnO (Al(5)) exhibits a significant difference in peak position compared to pristine and other co-doped ZnO samples. Moreover, the density of unoccupied states of Zn increases, suggesting the occurrence of charge transfer.

The crystal field splitting is examined through the energy difference between the orbitals t$_{2g}$ and e$_g$, and this separation is referred to as 10*Dq* (the energy of crystal field splitting). The value of 10*Dq* can be determined from the absorption spectrum of the complex. The t$_{2g}$ orbitals are 4*Dq* below the average energy level, while the e$_g$ orbitals are 6*Dq* above the average energy level. By using the equation E = $h$ $c$/$\lambda$ (where $\lambda$ represents the wavelength of the absorbing radiation, *h* denotes Planck’s constant, and *c* represents velocity of light), value of 10*Dq* can be determined. When the complex absorbs light with the appropriate wavelength, the electron is shifted from ground state (t$_{2g}$ orbital) to the excited state (e$_g$ orbital). The splitting of *d* orbitals in an octahedral crystal field is illustrated in Fig. S7 (supplementary text). Zn L-edge measurements revealing splitting of e$_g$ and t$_{2g}$ sub-bands by increasing the amount of Al doping. This is explained by considering the orbital hybridization in-between (Zn$^{2+}$) *d* and 2*p* (O atom) orbitals, orbital concepts, crystal field, and band anti-crossing (BAC) interaction^75,76^. The transition from bonding to antibonding states, induced by the BAC interaction of the absorbing electron, accounts for the splitting of e$_g$ and t$_{2g}$ sub-bands. The magnitude of 10*Dq* is influenced by several factors, such as the geometry of complexes, charge on metal ions, nature of ligands, and the position of the metal in the 1st, 2nd, or 3rd transition series. As a result, the degenerate set of *d* orbitals splits into two sets: e$_g$ orbitals with higher energy, including d$_{x^{2}-y^{2}}$ and d$_{z^{2}}$, and t$_{2g}$ orbitals with lower energy, including d$_{xy}$, d$_{yz}$, and d$_{zx}$ orbitals.

#### Cu K-edge NEXAFS

**Figure 11:**
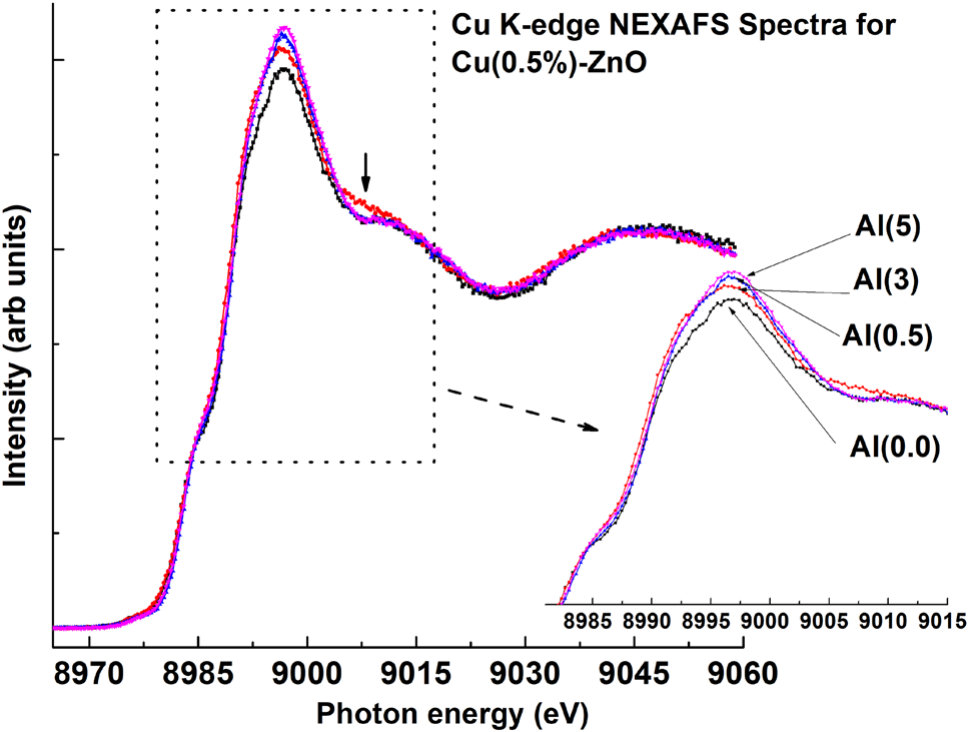
Normalized Cu K-edge NEXAFS spectra for Al$_x$Cu$_{0.5\%}$ZnO (x=0.0, 0.5%, 3%, and 5%) showing the variation with Al-doping in Cu(0.5%)-ZnO system.

The Cu K-edge NEXAFS of Al co-doped Cu-ZnO nanostructures and appearance of Cu$^{+2}$ state in Cu are illustrated in Fig. 11. The presence of a pre-edge hump at an energy of approximately 8997 eV (marked by downarrow) indicates a change in symmetry, which is further confirmed by the formation of nanorods observed in FESEM micrographs at higher Al doping levels.

**Figure 12:**
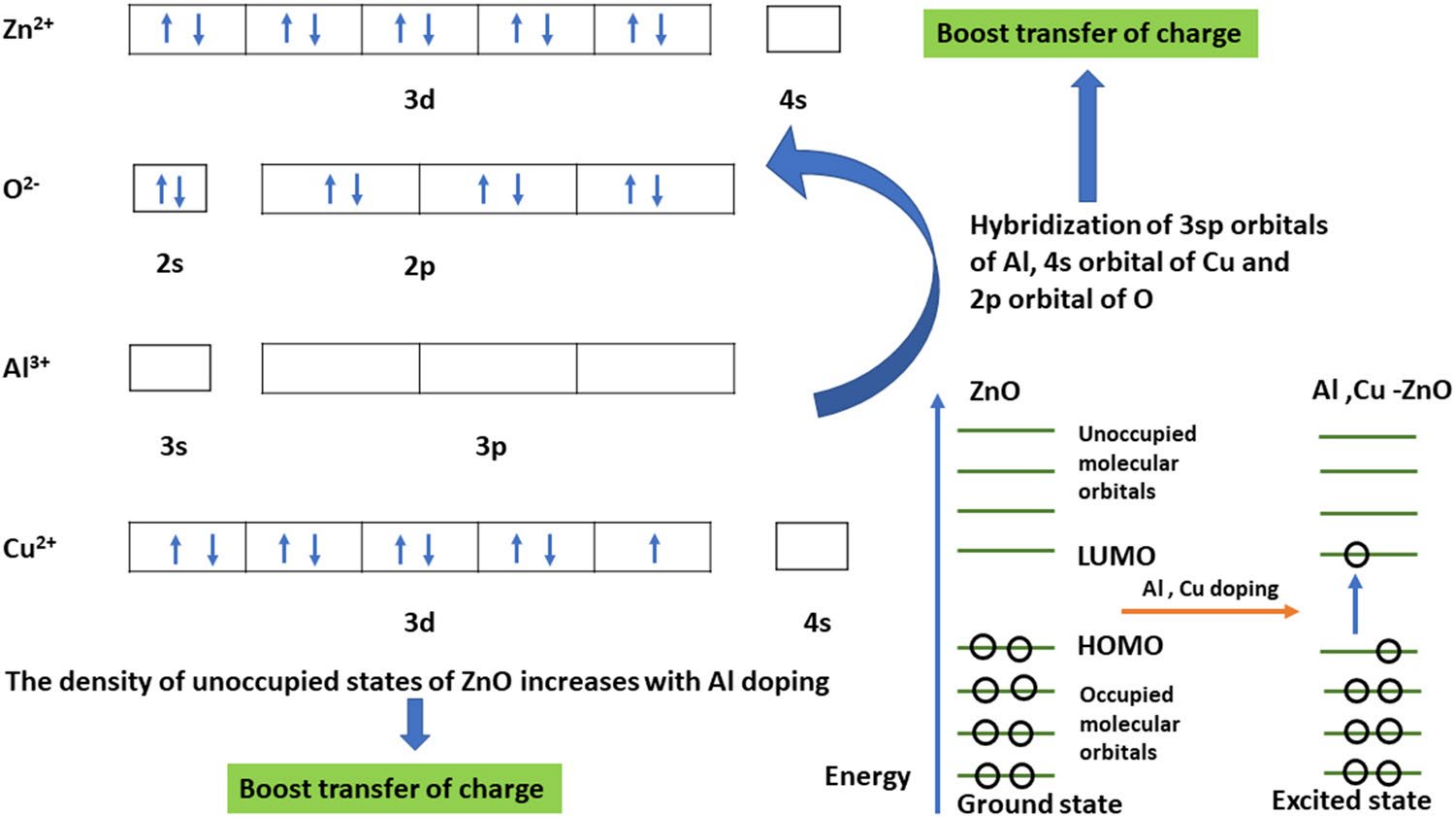
Charge transfer mechanism in Al co-doped Cu-ZnO nanostructure.

The intensity of main peak edge also increases after Al co-doping, ascribed to the concept of charge transfer. As the Al doping level increases, the density of unoccupied states increases, and more charge transfer occurs, as evidenced by the analysis of the Zn L-edge. The Cu 3$d^9$ 4$s^0$ orbital can facilitate the transfer of electrons from Al to Cu/Zn, considering that Al$^{3+}$ occupies the Zn$^{2+}$ site in the Zn matrix. Consequently, changes in the HOMO intensity^33^ and the transfer of charge from HOMO to LUMO occur. The phenomenon of charge transfer has also been discussed by Chen et al. regarding ferromagnetic properties^64,69^, as well as by Borgwardt et al. in the context of charge transfer dynamics following the emergence of interfacial electronic states at Dye-Sensitized zinc oxide interfaces^77^. The hybridization of the 3*sp* orbitals of incorporated Al with the 2*p* orbitals of O atoms promotes the transfer of charge from Al atoms to O atoms (Fig. 12). This concept can also be correlated with the results of O K-edge NEXAFS analysis.

### Photo sensitivity

**Figure 13:**
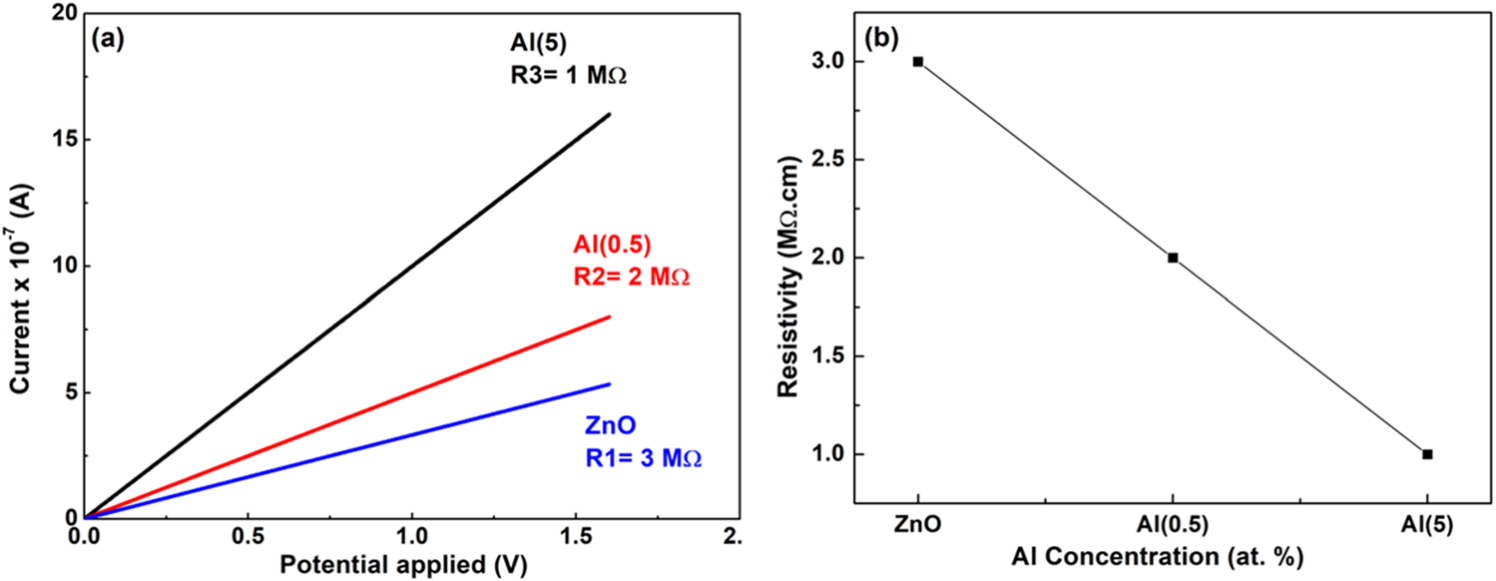
The photoilluminated current showing variation in resistivity of aluminium and copper co-doped zinc oxide nanostructure, (**a**) I-V plots, and (**b**) resistivity, for ZnO sample with Al doping concentration.

Figure 13 demonstrates the photoilluminated current of aluminium and copper co-doped zinc oxide nanostructures through I-V measurements. Using bias voltage of 1.5 V, the currents (photoilluminated) of pristine zinc oxide, Al(0.5), and Al(5) are observed as 0.5 $\mu$A, 0.7 $\mu$A, and 1.4 $\mu$A, respectively. The low current values indicate the appearance of good crystalline quality of nanostructures. Furthermore, current value linearly enhances with the applied voltage, demonstrating a good ohmic contact in samples. The resistance values of pristine zinc oxide, Al(0.5), and Al(5) co-doped zinc oxide are calculated as 3 M$\Omega$, 2 M$\Omega$, and 1 M$\Omega$, respectively. The zinc oxide nanostructure reveals *n*-type semiconductor behavior because of the presence of defects like zinc interstitial and oxygen vacancies^78^. With the increment of Al doping, the number of carrier concentration also increases, as Al carries one excess valence electron compared to Zn. The decrease in resistivity with up to 5% Al co-doping is attributed to the appearance of an free electron (extra) in the conduction band, originates with the doping of Al$^{3+}$ at Zn$^{2+}$ sites^79^ (Fig. 13b). Hence, after comparing the currents (photoilluminated), it can be observed that the aluminium and copper co-doped zinc oxide nanostructure exhibits improved photosensing capabilities.

### Conclusion

This research provides valuable insights into effects of Al co-doping on the electrical, optical, and structural properties of ZnO nanostructures. The substitution of Al ions leads to the emergence of (Cu,Al) co-doped zinc oxide samples with improved crystallinity, reduced band gap, and enhanced sensing capabilities. The increase in grain size, reduction of visible emission defects, and enhancement of O/Zn stoichiometry contribute to the enhanced photosensing performance in photodetectors. The PL and NEXAFS spectroscopy analyses confirm the appearance of charge transfer processes and creation of electron traps, further improving optical properties of the co-doped ZnO, and hence the improved photosensing. This study highlights potential of Al doping as a viable strategy for advancing the performance of photodetectors/solar cell electrodes, offering a promising avenue for future applications that require transparent, durable, cost-effective, and portable sensing devices.

## Supplementary Information

Supplementary Information.

## Data Availability

The crystallographic data generated is available in the Supplementary file. The other data may be provided on request from corresponding author Sanjeev Gautam (sgautam@pu.ac.in).
