# Supplementary material for: Charge transfer and X-ray absorption investigations in aluminium and copper co-doped zinc oxide nanostructure for perovskite solar cell electrodes
Source: Sci Rep. 2023 Jul 4;13:10769. doi: 10.1038/s41598-023-37754-1 (PMC10319901; doi:10.1038/s41598-023-37754-1)
Supplement: Supplementary file 1 — Supplementary Information. [file 41598_2023_37754_MOESM1_ESM.docx]

# Charge transfer and X-ray absorption investigations in aluminium and copper co-doped zinc oxide nanostructure for perovskite solar cell electrodes

# Mandeep Kaur^1,4^, Sanjeev Gautam^1,*^, Keun Hwa Chae^2^, Wantana Klysubun^3^, and Navdeep Goyal^4^

^1^Advanced Functional Materials Lab., Dr. S. S. Bhatnagar University Institute of Chemical Engineering & Technology, Panjab University, Chandigarh, 160 014, India

^2^Advanced Analysis Center, Korea Institute of Science and Technology, Seoul, South Korea

^3^Synchrotron Light Source Research Institute, Nakhon Ratchasima 30000, Thailand

^4^Department of Physics, Panjab University, Chandigarh 160 014, India

Corresponding Author Email: [sgautam@pu.ac.in](mailto:sgautam@pu.ac.in)

## X-ray diffraction Analysis

**Table T1 : Crystallite size variation to the (101) plane with different dopant materials.**

| Sample Name  (ZnO-dopant%) | (101)-  2*θ (in deg.)* | Crystallite  size(nm) - D |
| --- | --- | --- |
| Pristine ZnO | 36.34 | 44 |
| ZnO-Cu(0.5%) | 36.28 | 61 |
| ZnO-Al(0.5%) | 36.28 | 50 |
| ZnO-Cu(0.5%)-Al(0.5%) | 36.28 | 56 |
| ZnO-Cu(0.5%)-Al(1%) | 36.28 | 78 |
| ZnO-Cu(0.5%)-Al(3%) | 36.32 | 90 |
| ZnO-Cu(0.5%)-Al(5%) | 36.28 | 83 |


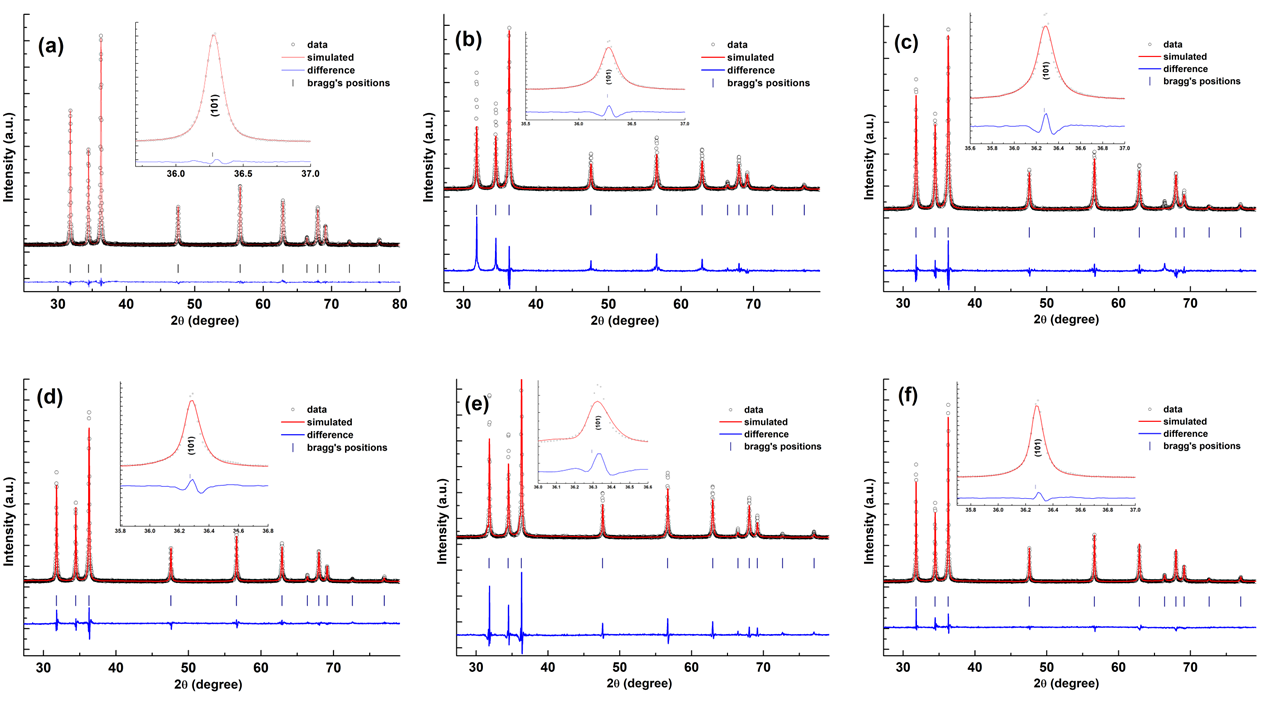


**Figure S1: Rietveld refinement of X-ray diffraction data for (a) pristine ZnO, (b) Cu(0.5), (c) CuAl(0.5), (d) Al(1), (e) Al(3), and (f) Al(5) using P63mc space group. Open circles correspond to the X-ray diffraction data and the lines are theoretical fits to the observed X-ray data. Vertical bars are the Bragg reflections for the space group. The difference pattern between the observed data and the theoretical fit is shown in the bottom. (The inset : enlarged view of pristine ZnO (101) peak).**

Figure S1: Rietveld refinement of pristine ZnO.

**Fourier Transform Infrared Spectroscopy Analysis**


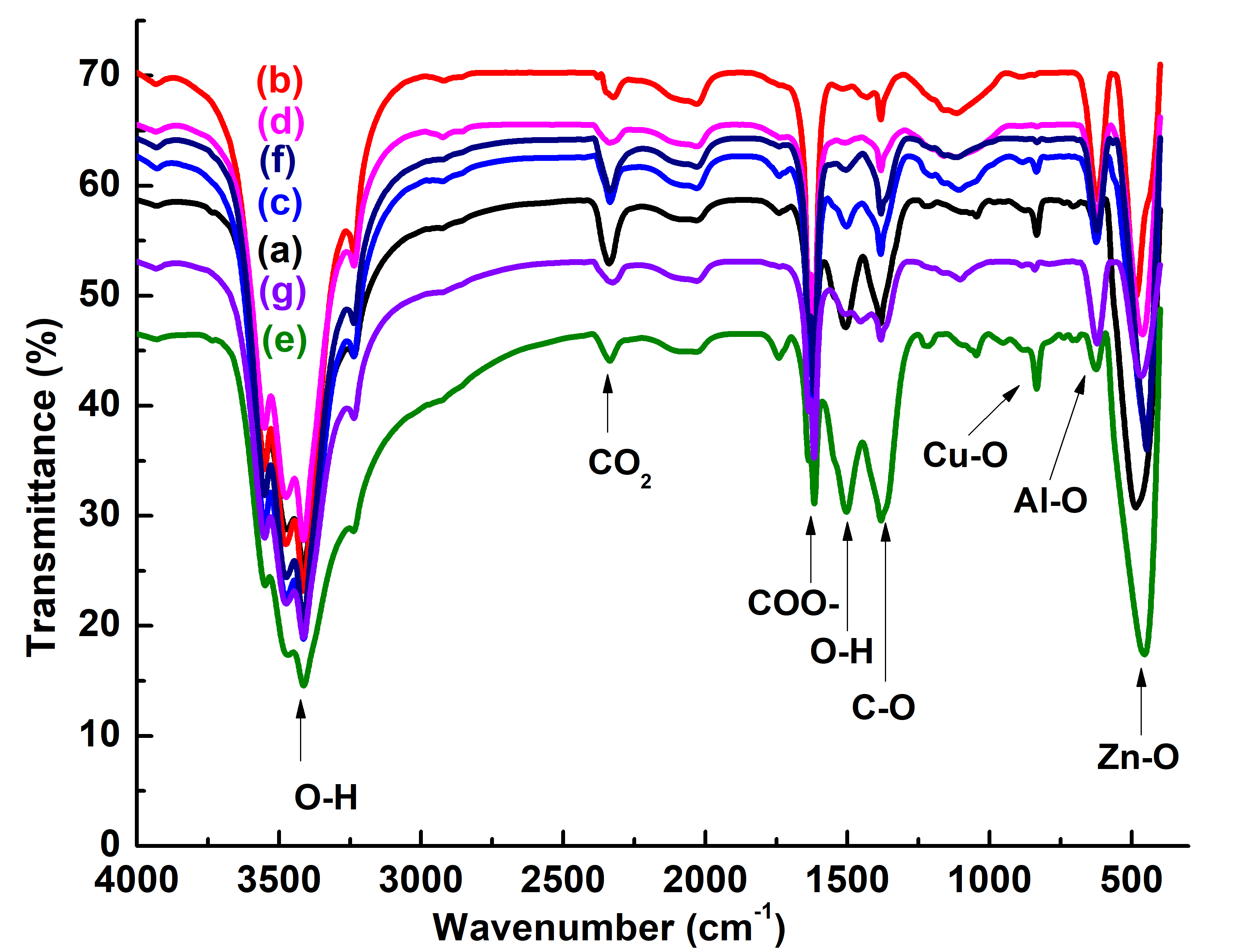


**Figure S 2: Fourier Transform Infrared Spectra of (a) ZnO-P, (b) ZnO-Cu(0.5%), (c) ZnO-Al(0.5%), (d) ZnO-Cu(0.5%)-Al(0.5%), (e) ZnO-Cu(0.5%)-Al(1%), (f) ZnO-Cu(0.5%)-Al(3%), and (g) ZnO-Cu(0.5%)-Al(5%).**

## Photoluminescence spectrum analysis

**Table T 2: Table of different peak position (wavelength) and FWHM values of Al and Cu co-doped ZnO samples.**

|  | **Zn1 (nm)** | **Zn2 (nm)** | **Zn3 (nm)** | **Zn4 (nm)** | **Zn5 (nm)** |
| --- | --- | --- | --- | --- | --- |
| **Pr** | 365.71 | 406.63 | 429.26 | 455.59 | 466.78 |
| **Cu(0.5)** | 365.82 | 406.75 | 429.52 | 456.98 | 465.97 |
| **Al(0.5)** | 366.11 | 409.33 | 430.88 | 447.42 | 460.78 |
| **CuAl(0.5)** | 365.82 | 406.76 | 429.48 | 456.78 | 466.18 |
| **Al(1)** | 366.01 | 406.49 | 428.93 | 455.47 | 461.19 |
| **Al(3)** | 365.97 | 406.68 | 429.29 | 455.93 | 465.69 |
| **Al(5)** | 365.96 | 407.03 | 428.85 | 451.54 | 455.88 |
| **FWHM Values** | | | | | |
| **Pr** | 14.07 | 18.83 | 27.02 | 30.24 | 74.81 |
| **Cu(0.5)** | 14.05 | 18.75 | 28.68 | 29.69 | 75.93 |
| **Al(0.5)** | 14.05 | 21.74 | 20.25 | 26.81 | 48.69 |
| **CuAl(0.5)** | 14.05 | 18.78 | 28.51 | 30.08 | 76.06 |
| **Al(1)** | 14.11 | 18.18 | 30.31 | 30.13 | 84.16 |
| **Al(3)** | 14.15 | 18.73 | 27.44 | 31.54 | 78.08 |
| **Al(5)** | 14.03 | 18.67 | 26.91 | 32.41 | 75.98 |

##
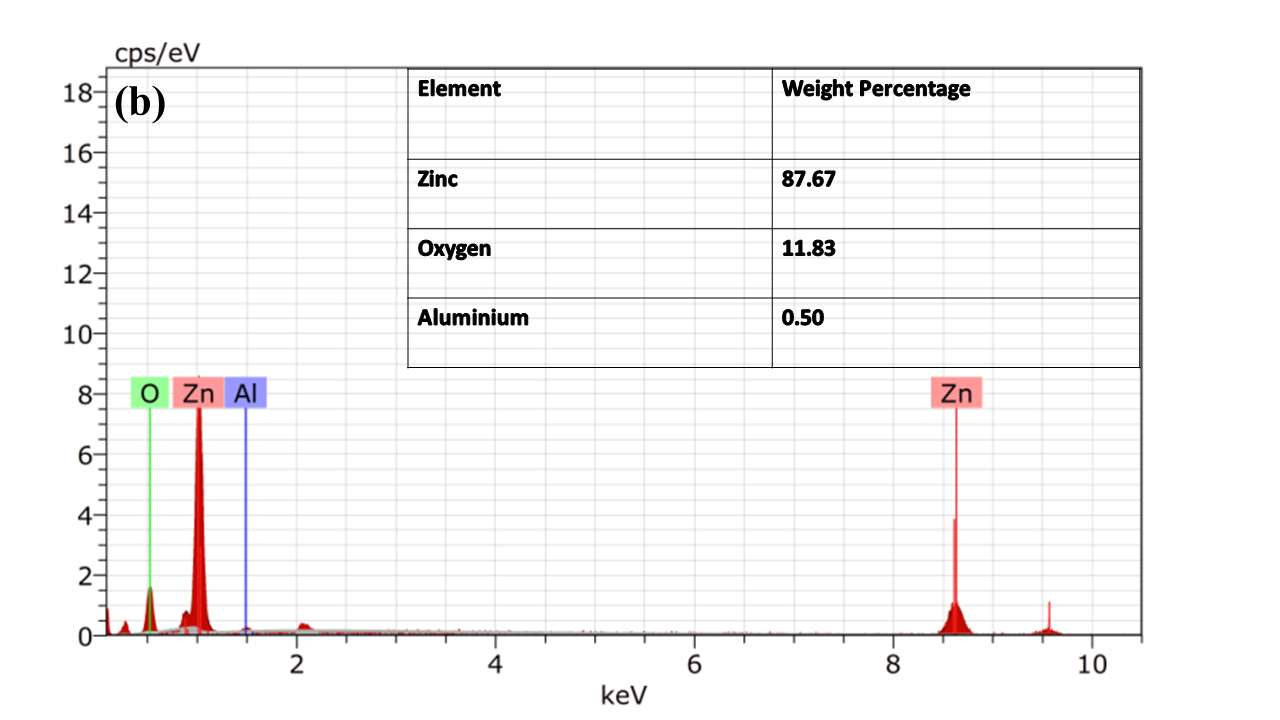

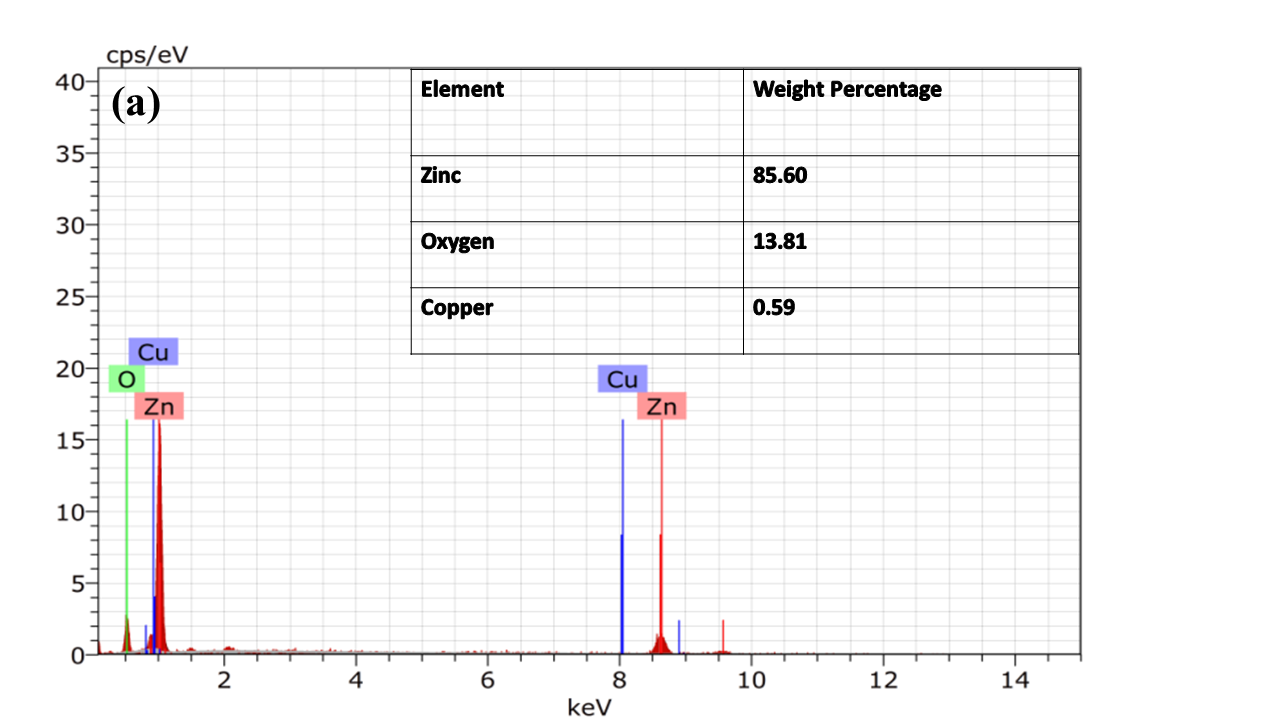
The Energy Dispersive X-ray (EDX) microanalysis analysis

**Figure S4: The Energy Dispersive X-ray spectra of Al(0.5) nanoparticles.**

**Figure S3: The Energy Dispersive X-ray spectra of Cu(0.5) nanoparticles.**


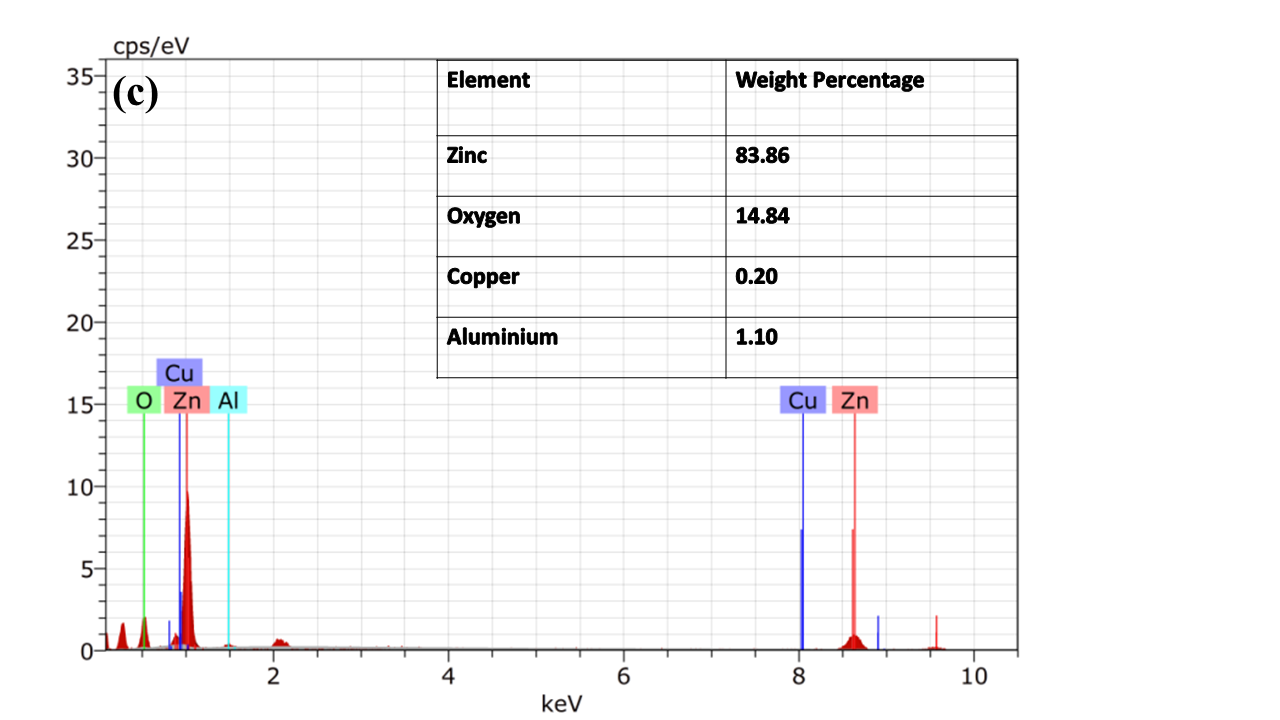


**Figure S5: The Energy Dispersive X-ray spectra of Al(1) nanoparticles.**

**
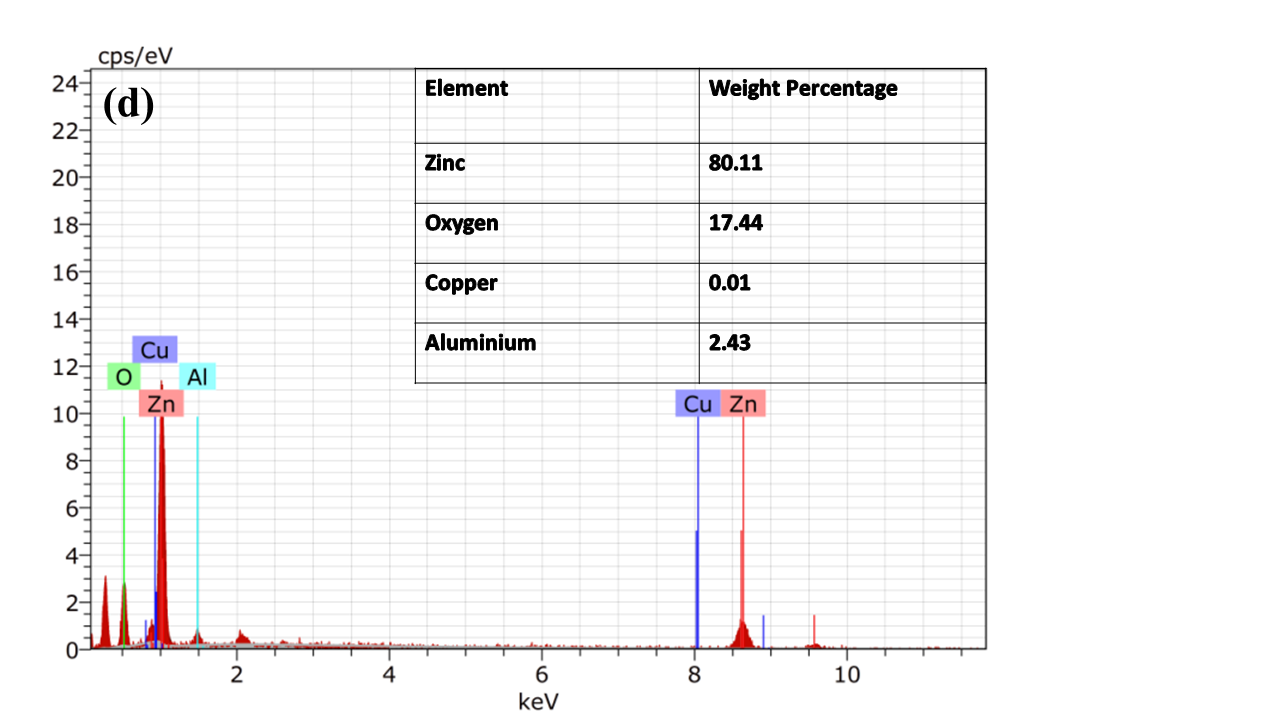
**

**Figure S6: The Energy Dispersive X-ray spectra of Al(5) nanoparticles.**

## Crystal field splitting energy (10 Dq)


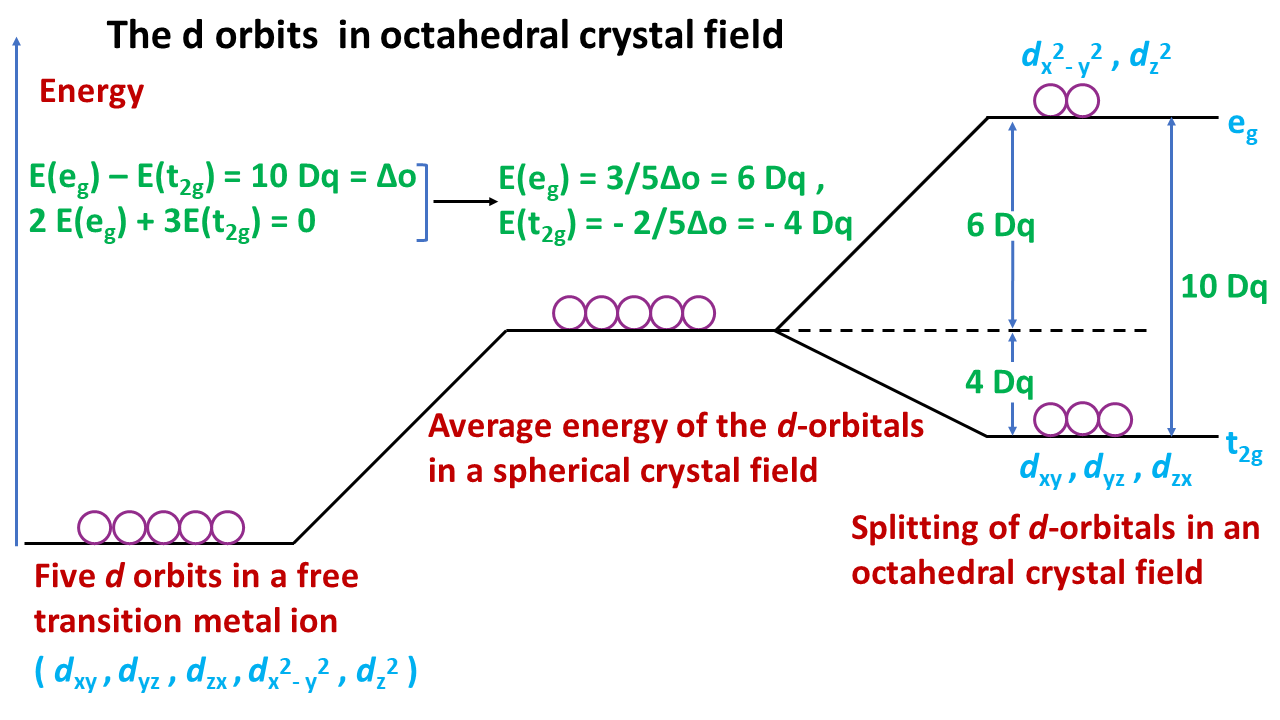


**Figure S7: Splitting of d-orbitals in an octahedral crystal field.**

## Charge transfer mechanism and concept of hybridization

##
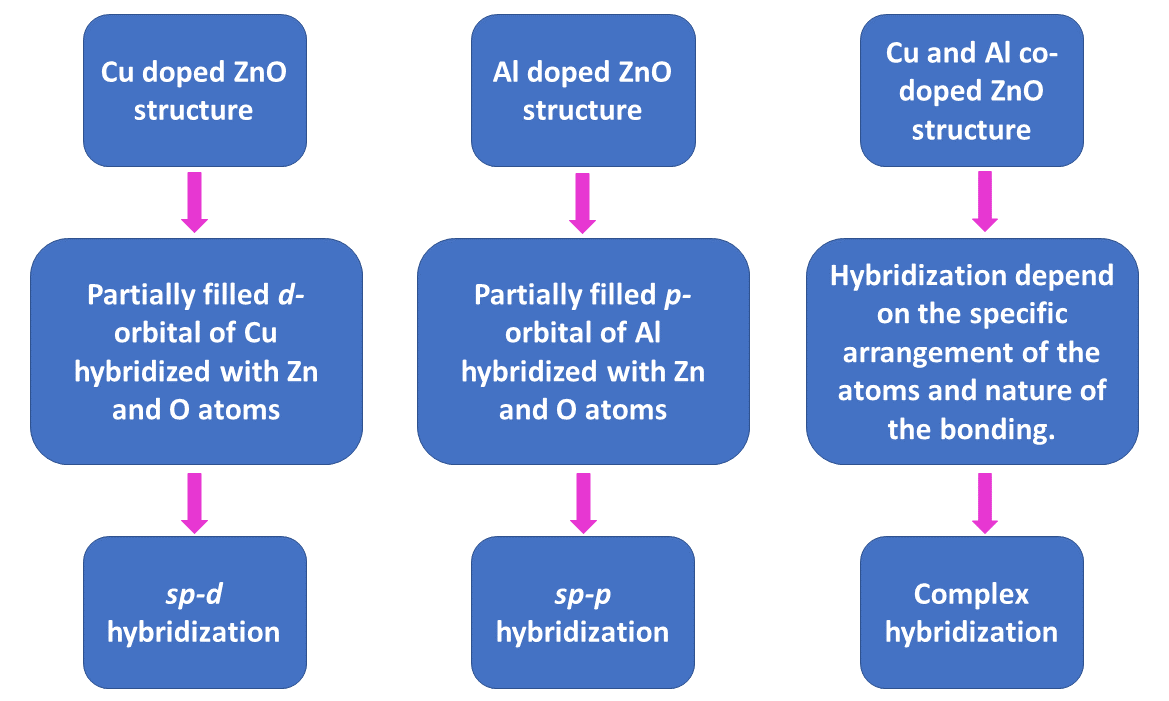


**Figure S8: Flowchart diagram showing hybridization of Al and Cu co-doped ZnO structure.**

**Enhanced photosensing mechanism**


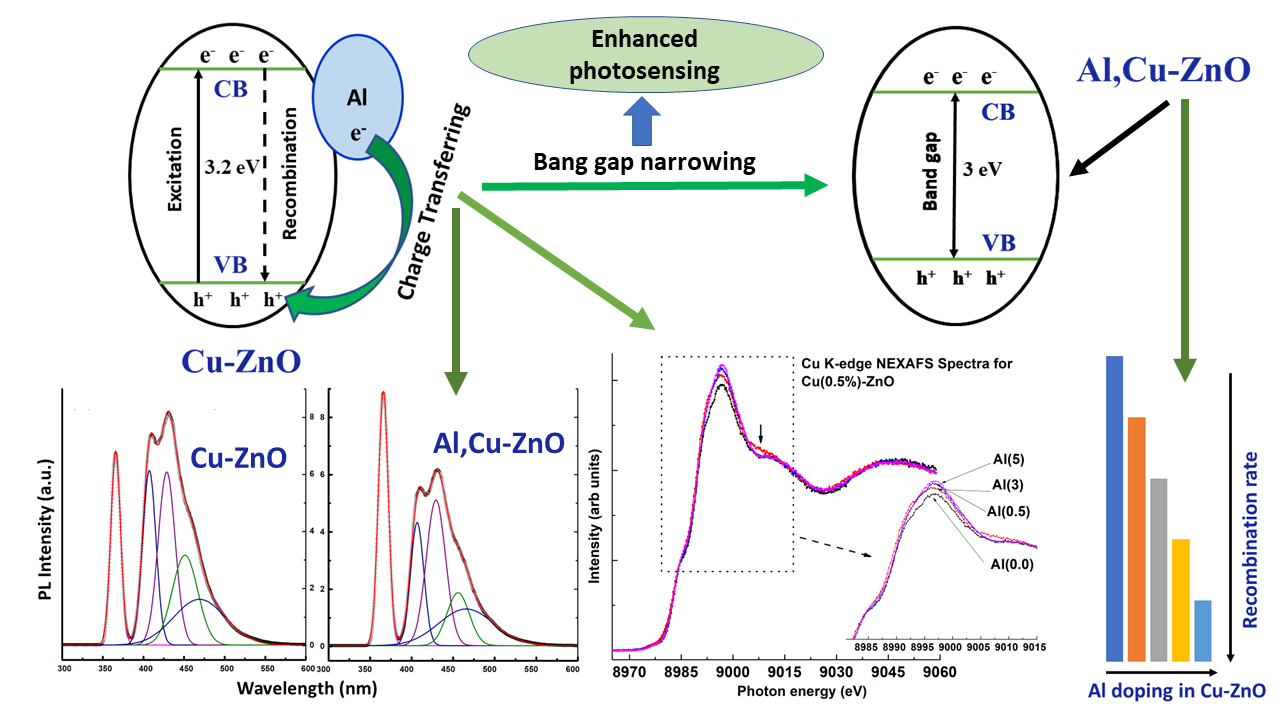


**Figure S9: Enhanced photo-sensing explanation through NEXAFS, PL, and Band gap narrowing.**

## Photoluminescence spectra (PL) analysis


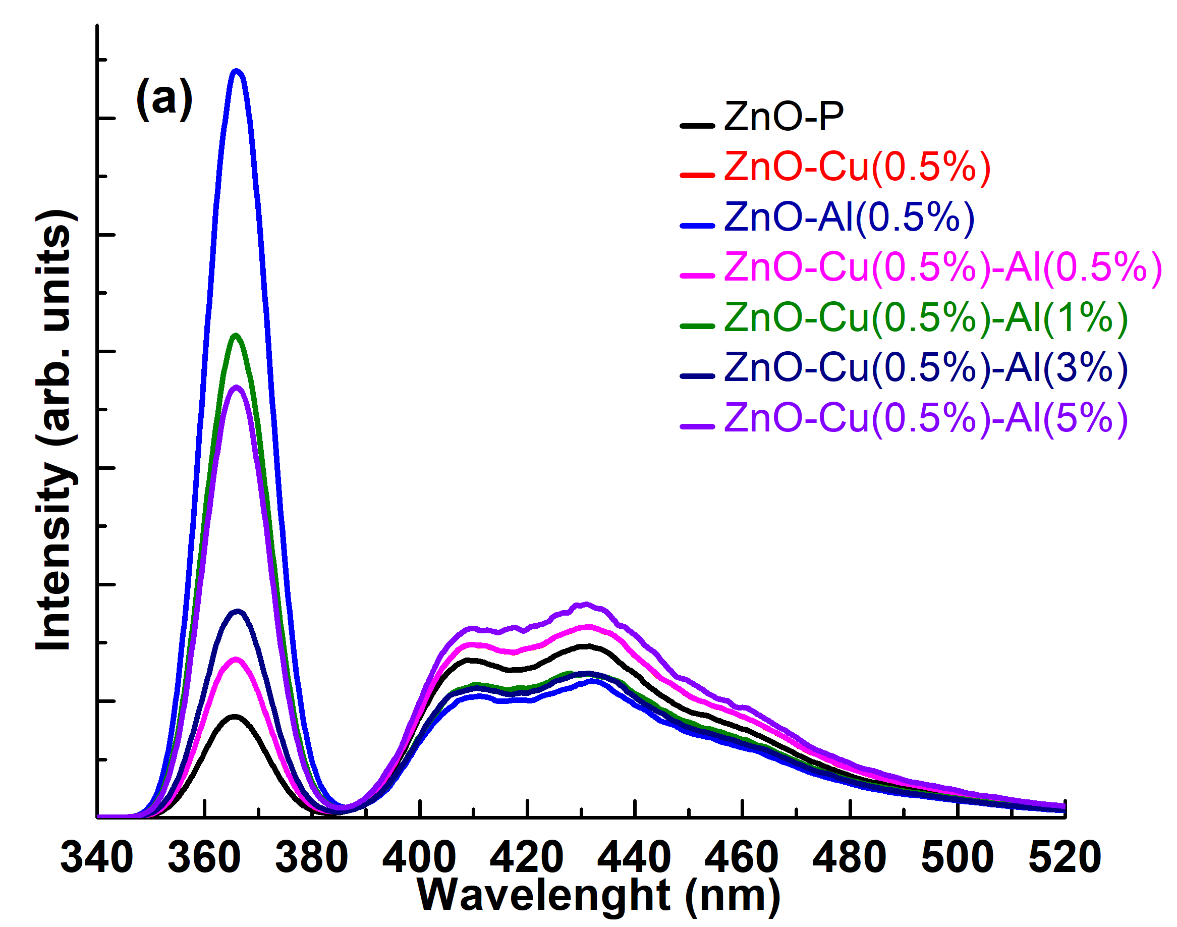


**Figure S10 : Photoluminescence spectra (PL) analysis of (a) ZnO-P, (b) ZnO-Cu(0.5%), (c) ZnO-Al(0.5%), (d) ZnO-Cu(0.5%)-Al(0.5%), (e) ZnO-Cu(0.5%)-Al(1%), (f) ZnO-Cu(0.5%)-Al(3%), and (g) ZnO-Cu(0.5%)-Al(5%).**

**Stress analysis (W-H plots)**

| 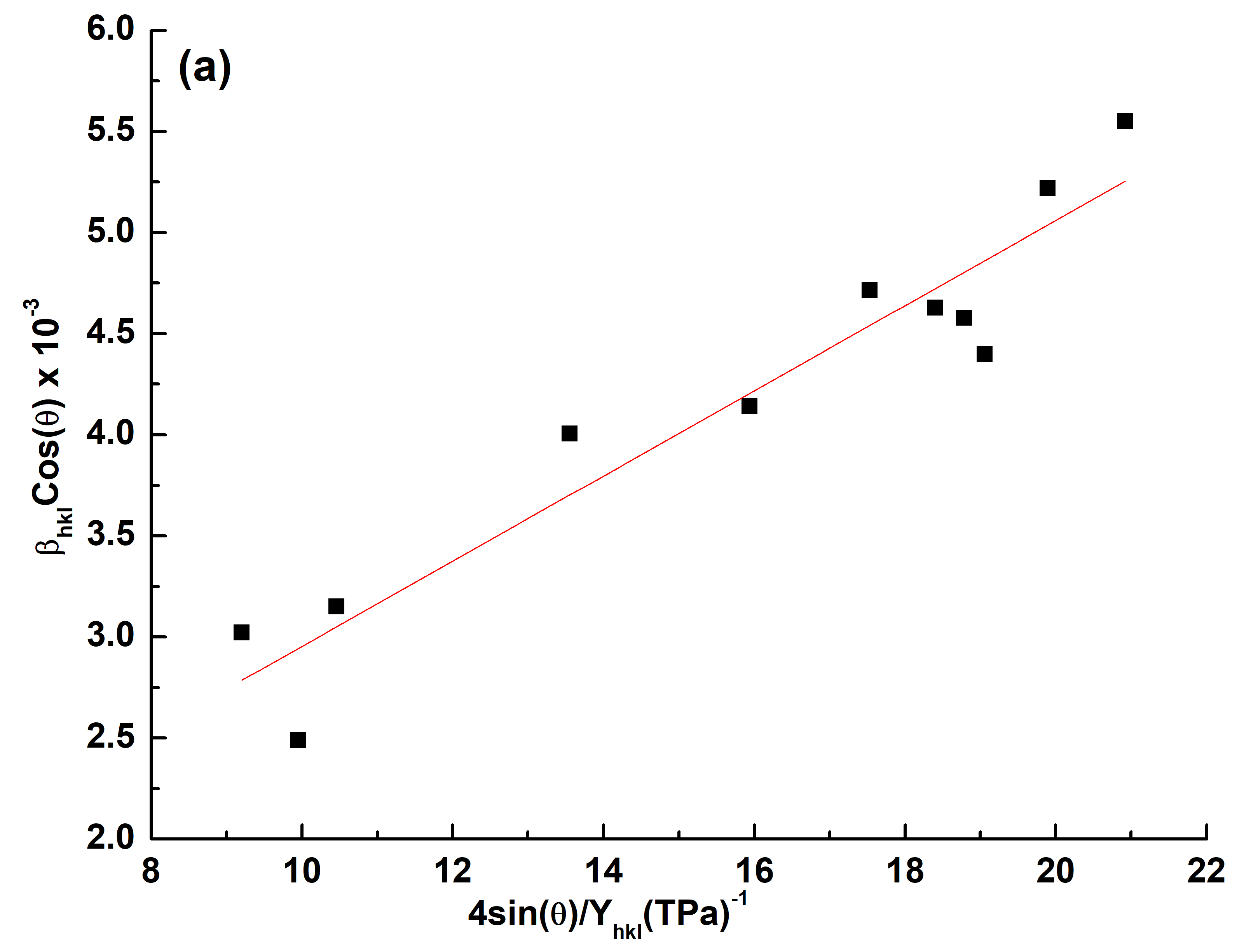 | 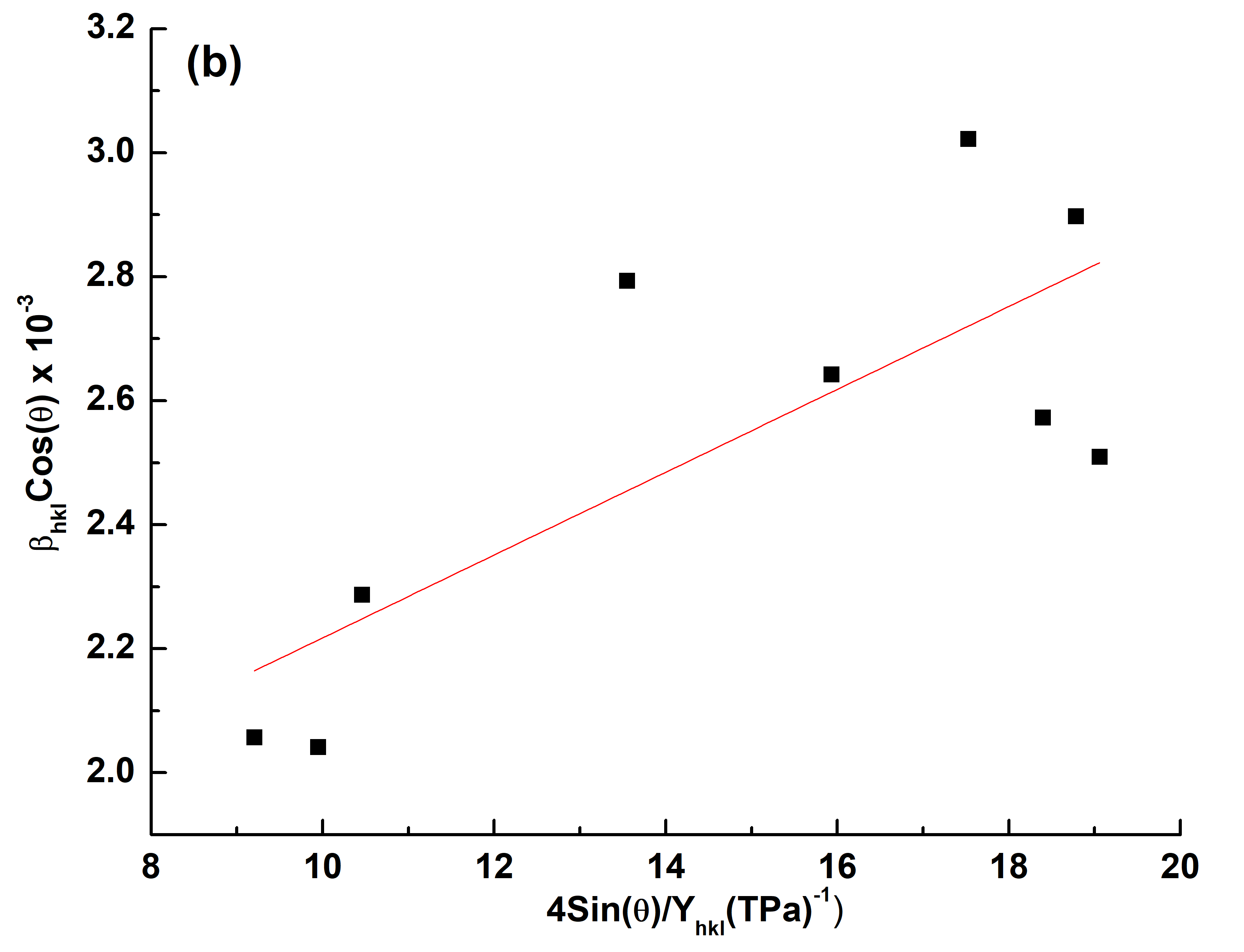 |
| --- | --- |
| 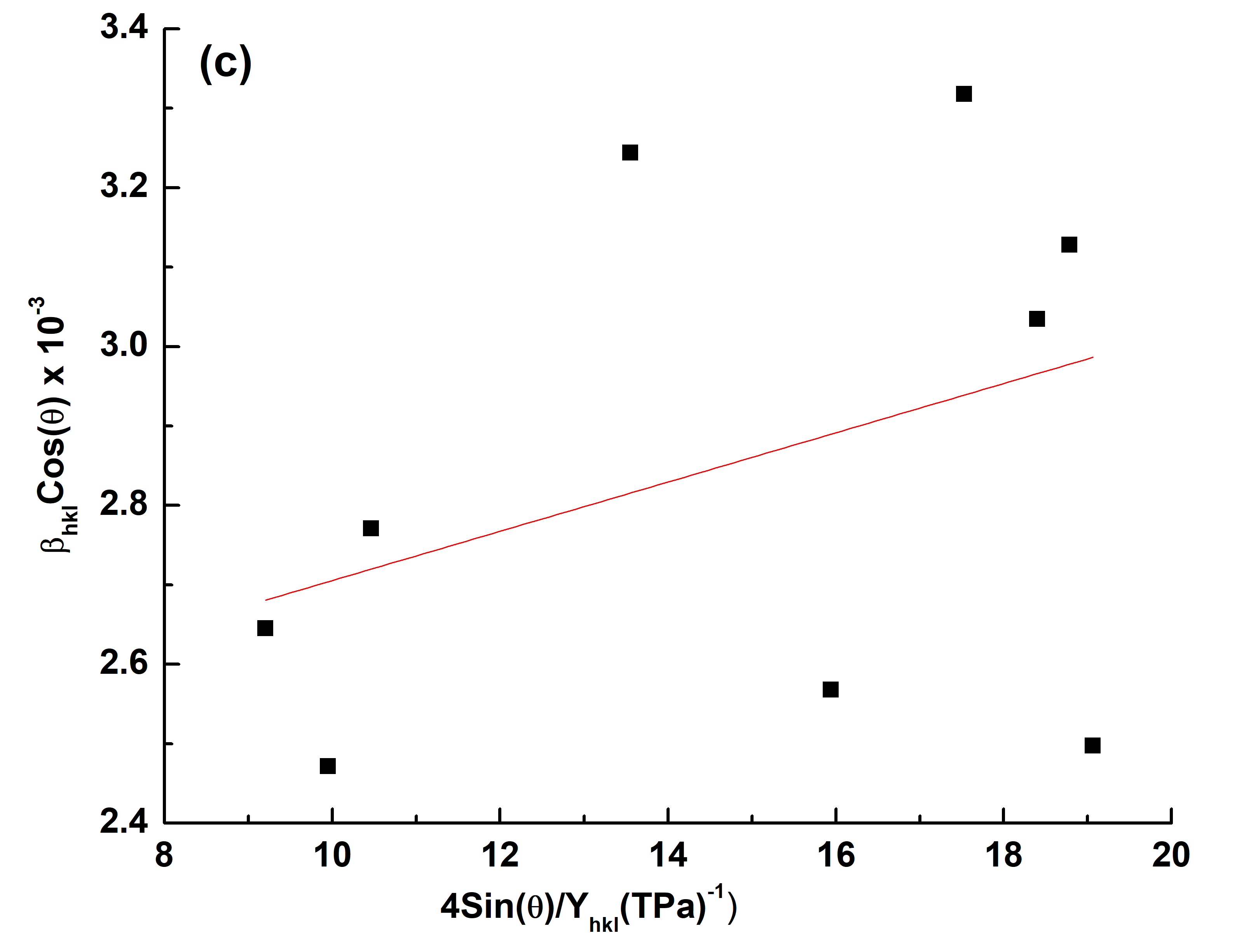 | 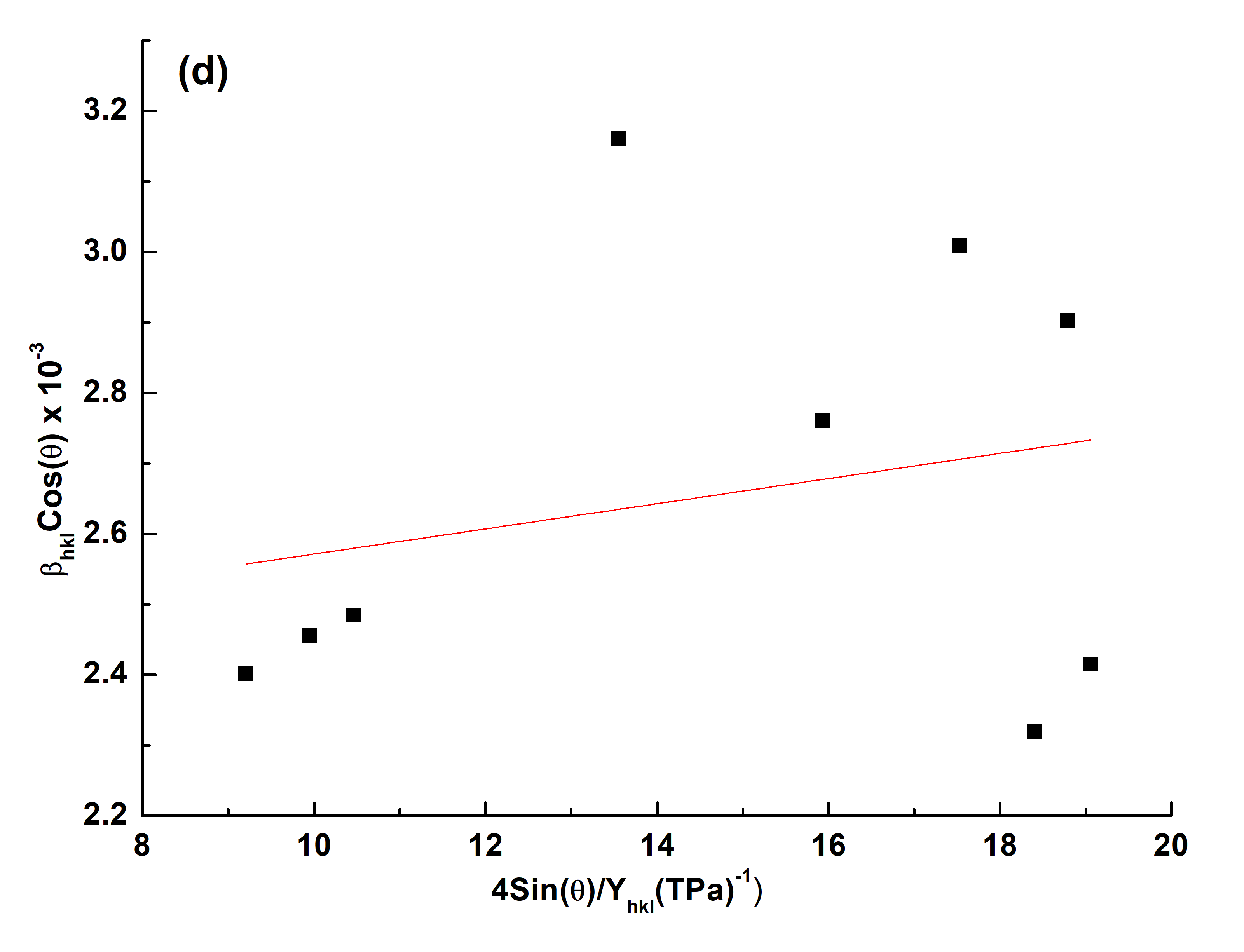 |

**Figure S11: W-H plots with linear fitting based on USDM for stress approximation a) pristine ZnO, (b) Cu(0.5), (c) Al(0.5), and (d) CuAl(0.5).**

| 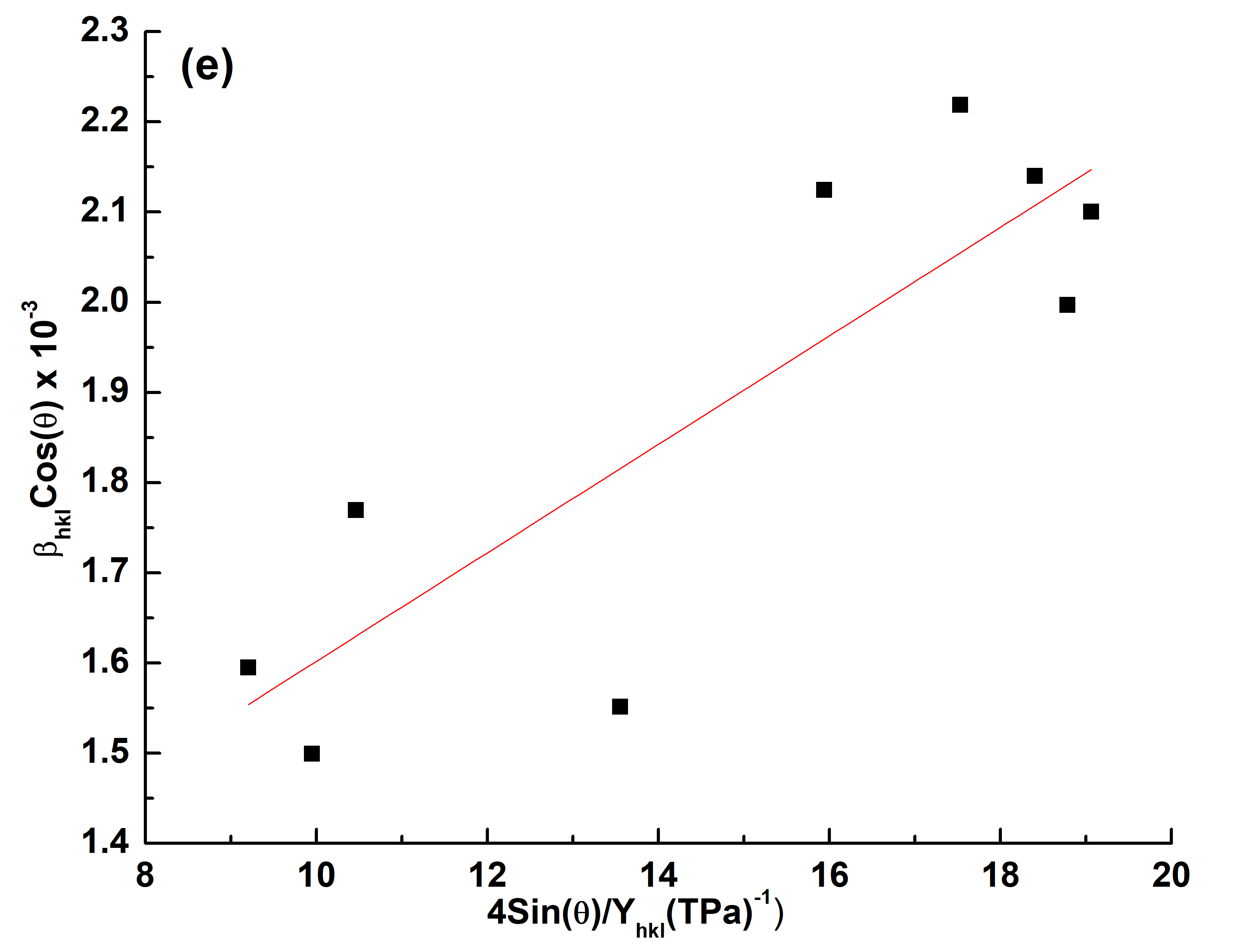 | 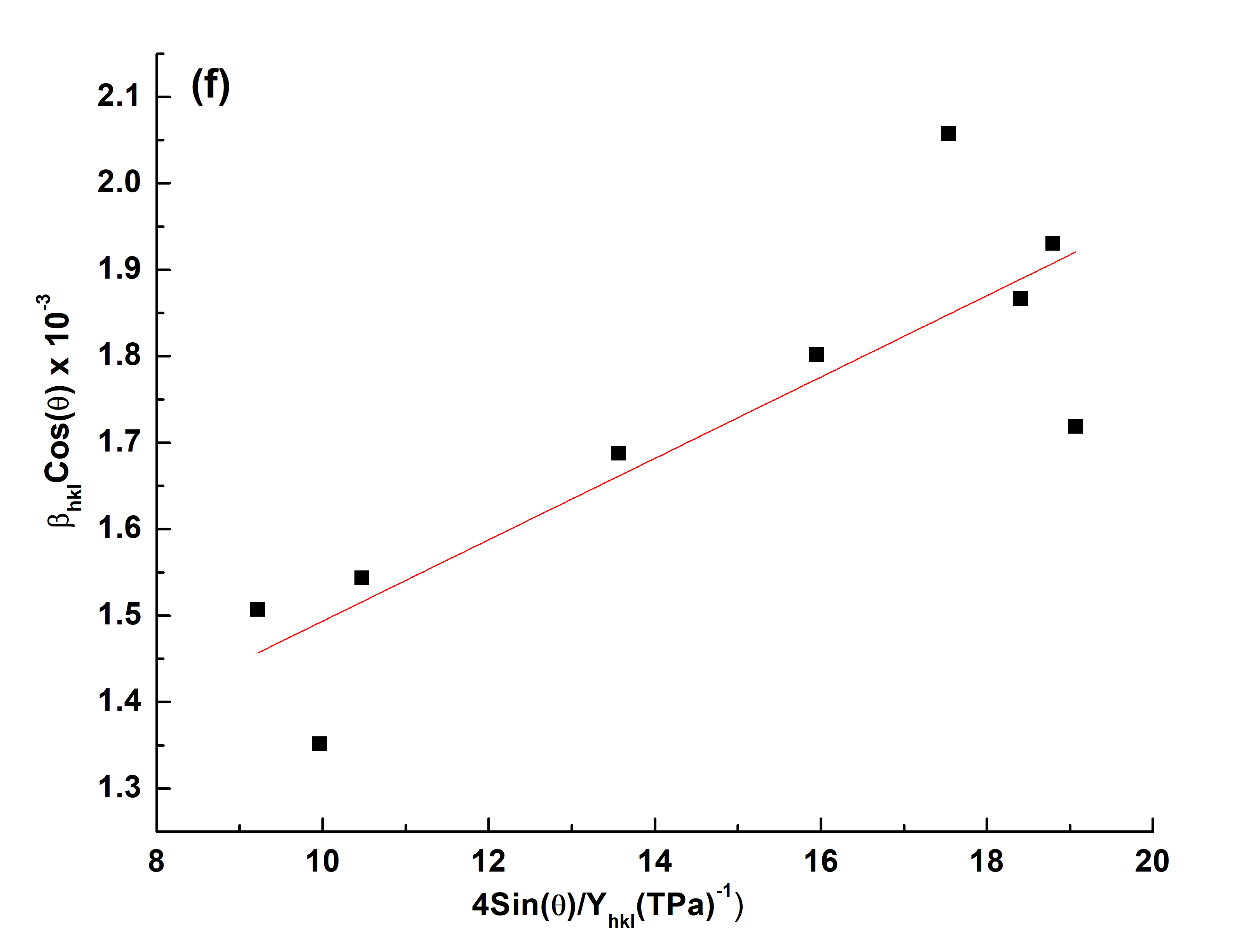 |
| --- | --- |
| 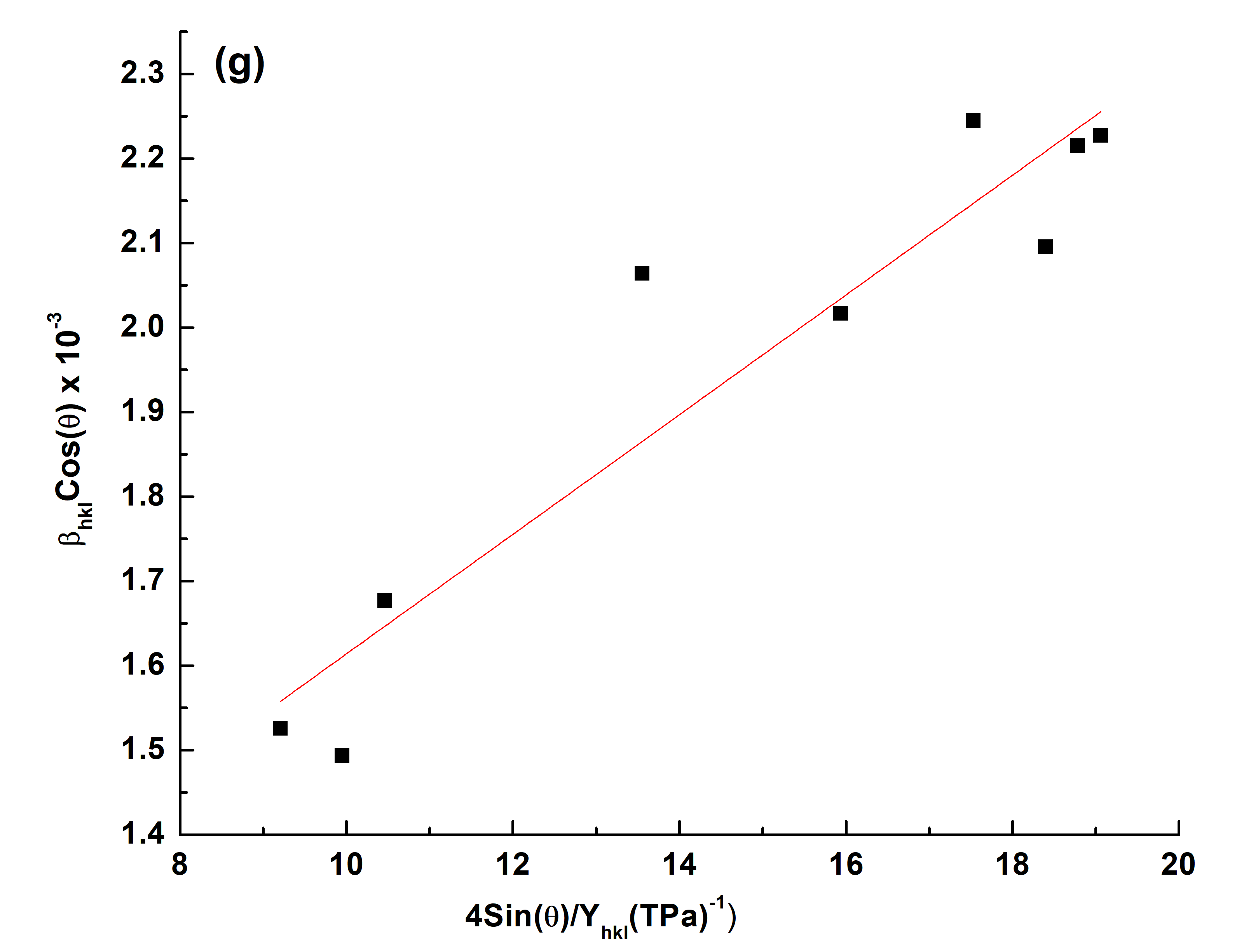 | 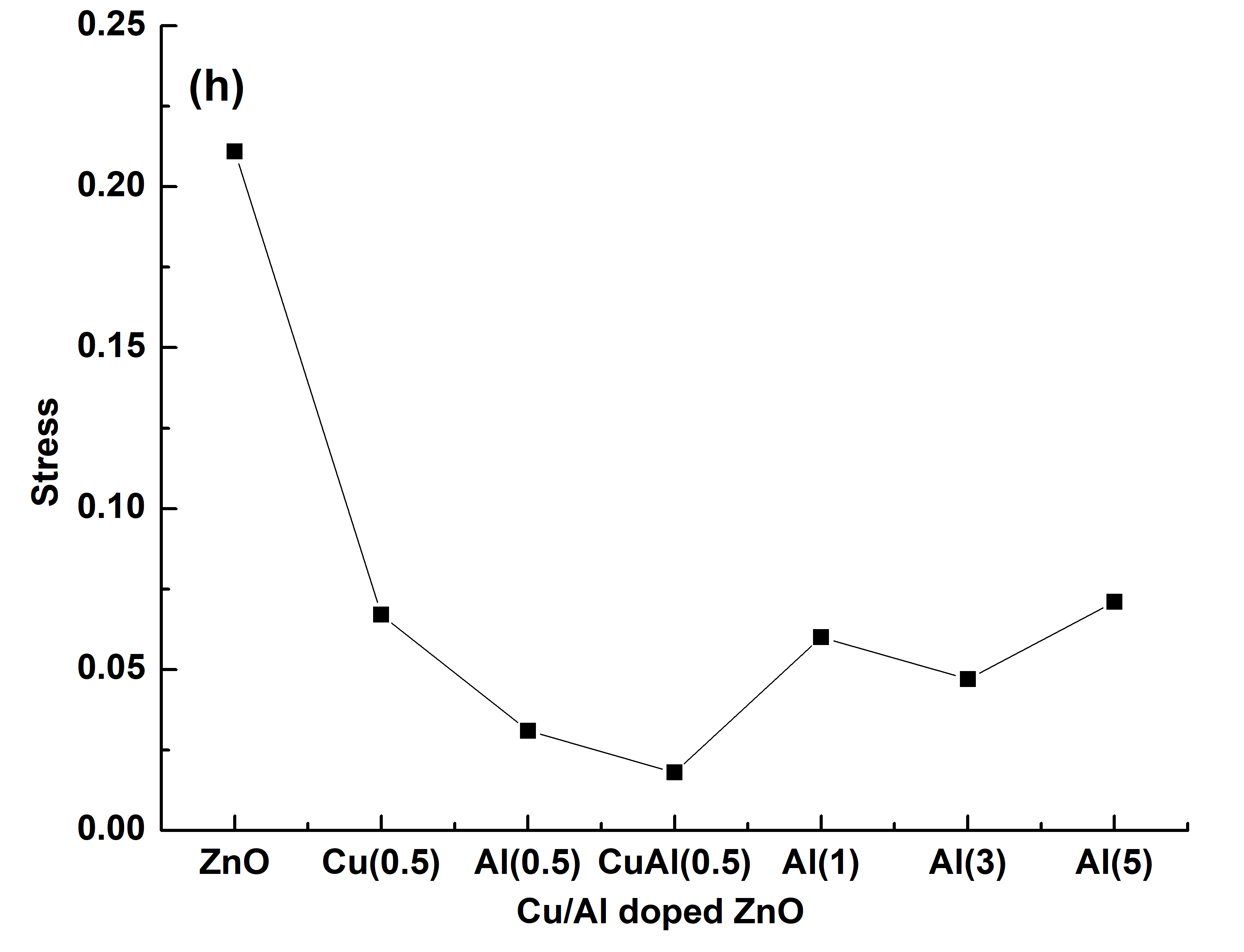 |

**Figure S12: W-H plots with linear fitting based on USDM for stress approximation (e) Al(1), (f) Al(3), (g) Al(5), and (h) behavior of stress value with increasing Al dopant .**

**Strain analysis (W-H plots)**

| 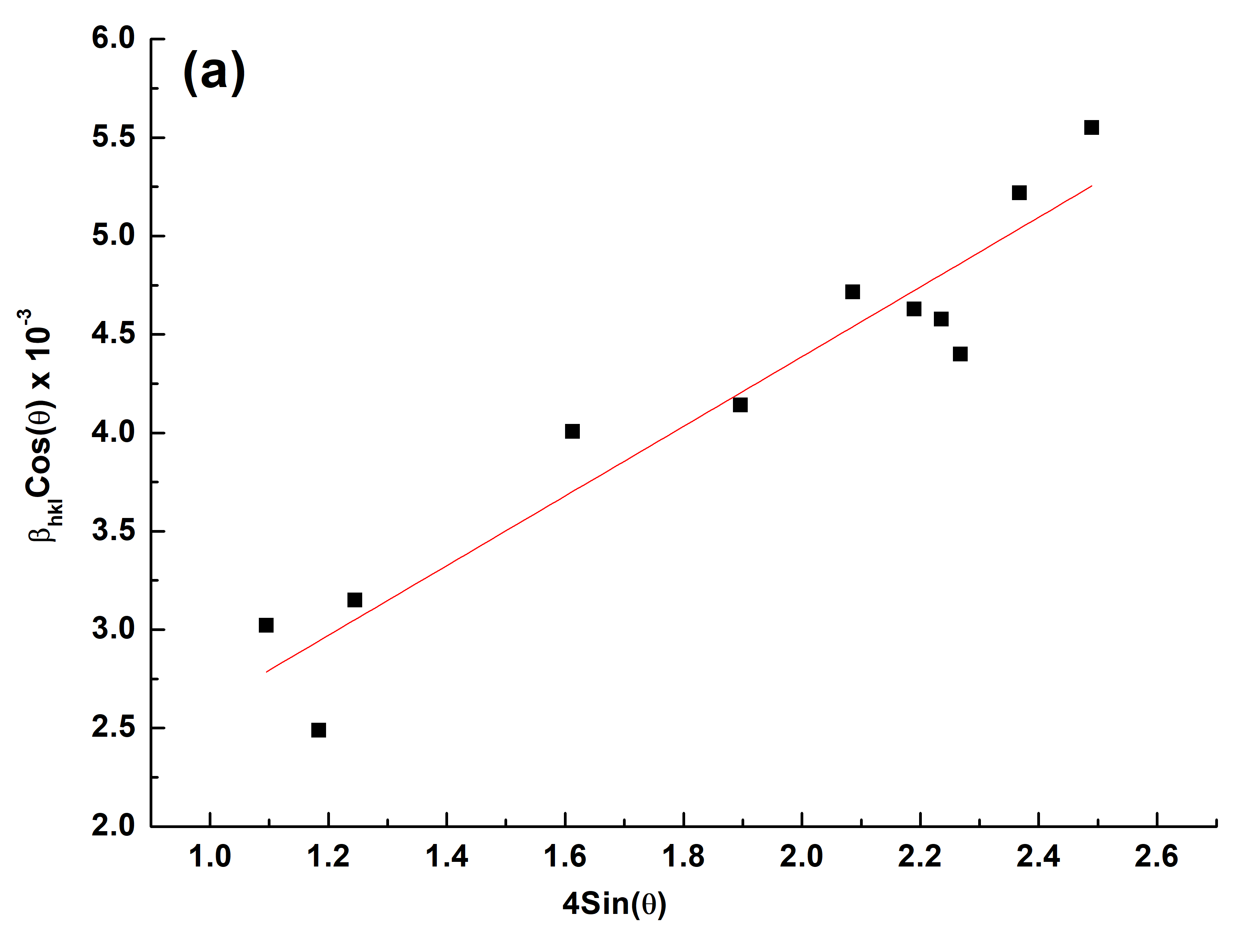 | 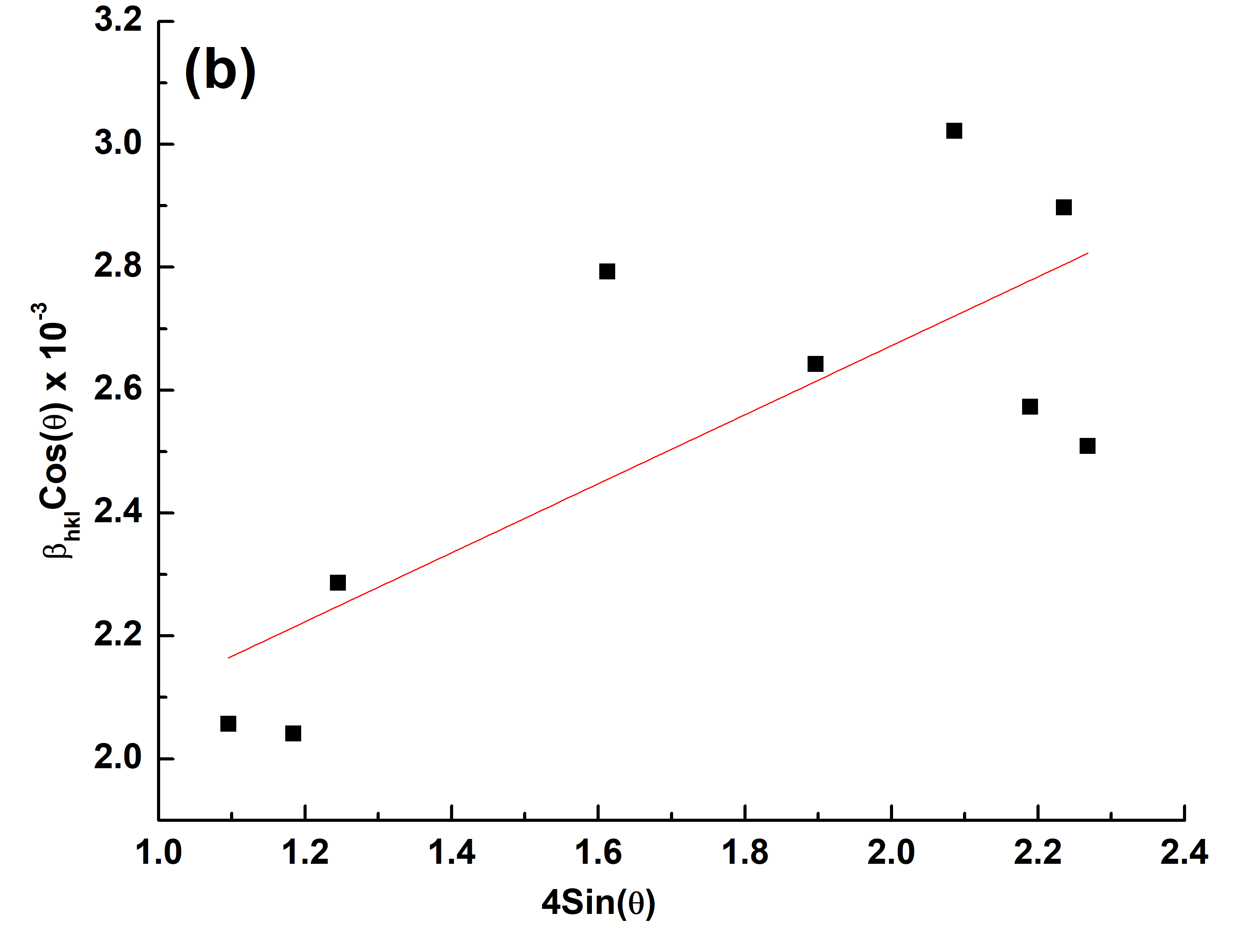 |
| --- | --- |
| 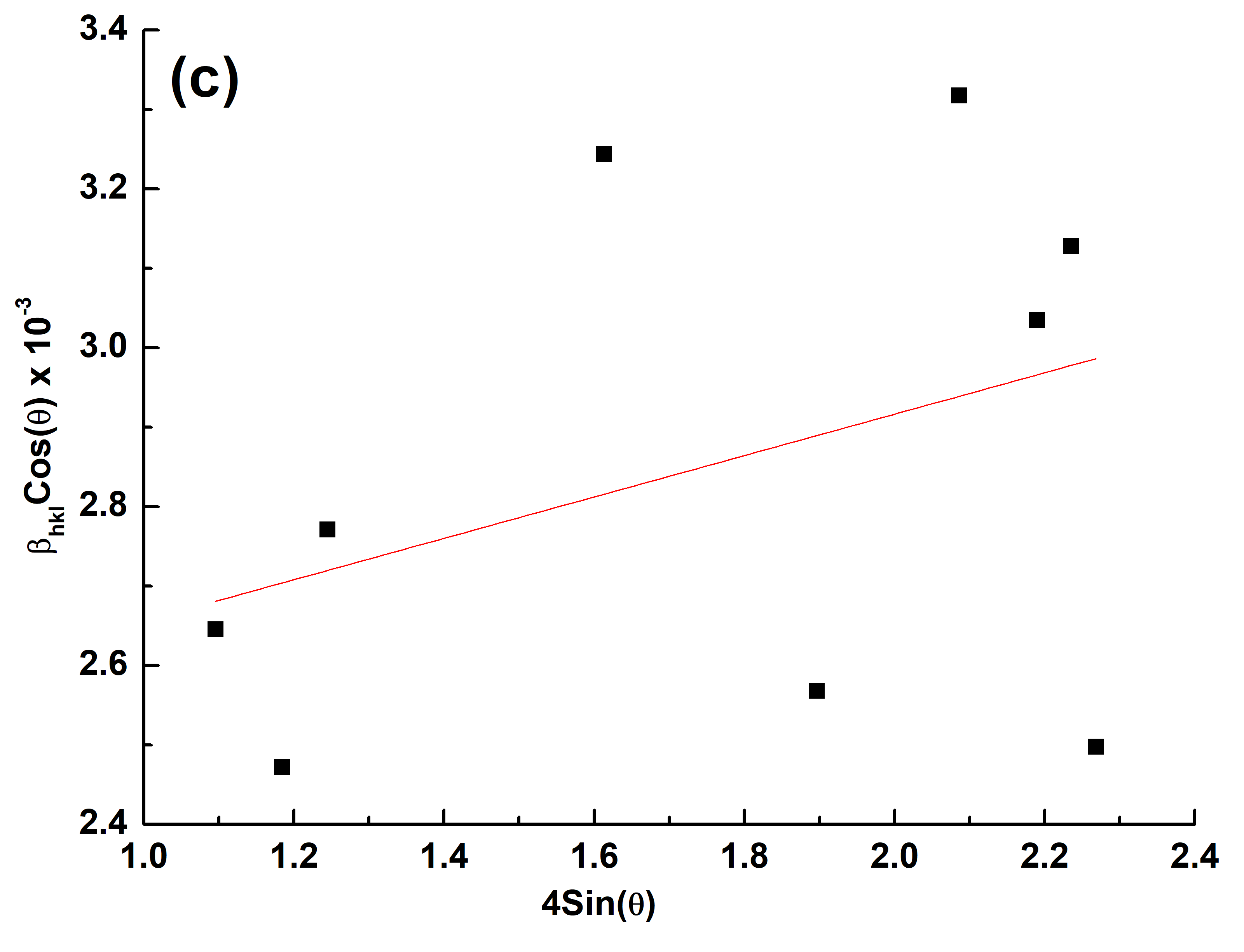 | 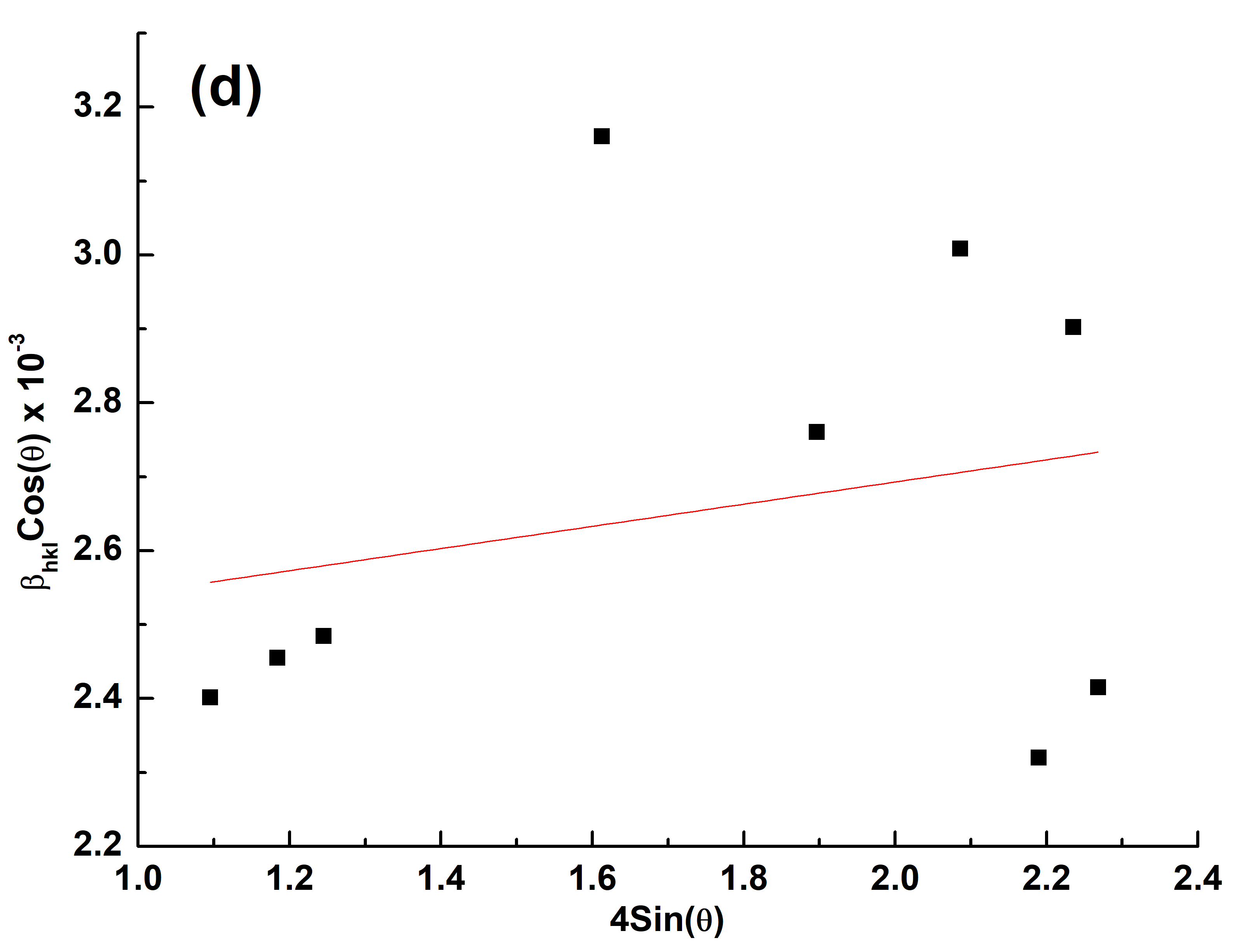 |

***Figure S13: W-H plots with linear fitting based on UDM for strain approximation a) pristine ZnO, (b) Cu(0.5), (c) Al(0.5), and (d) CuAl(0.5).***

| 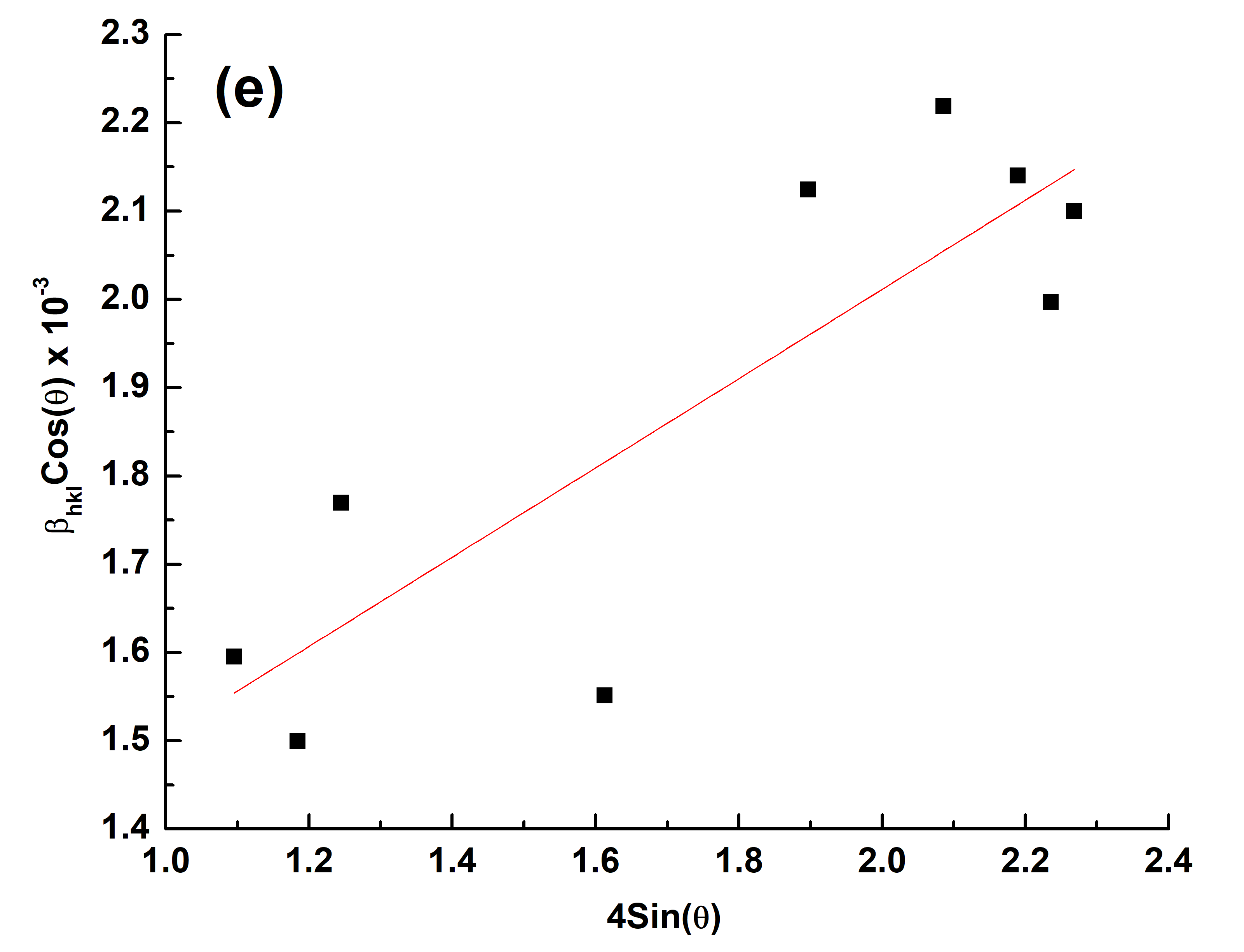 | 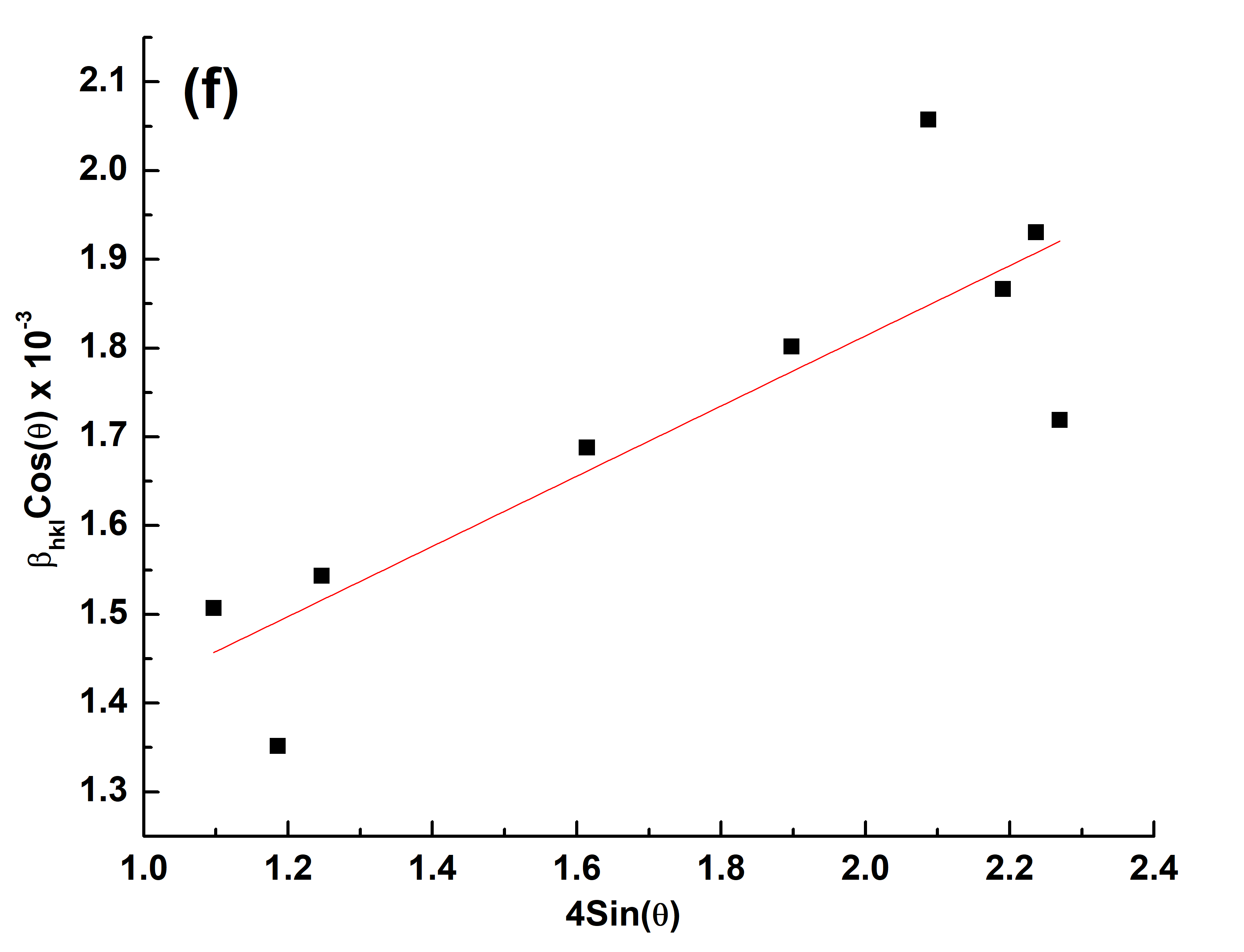 |
| --- | --- |
| 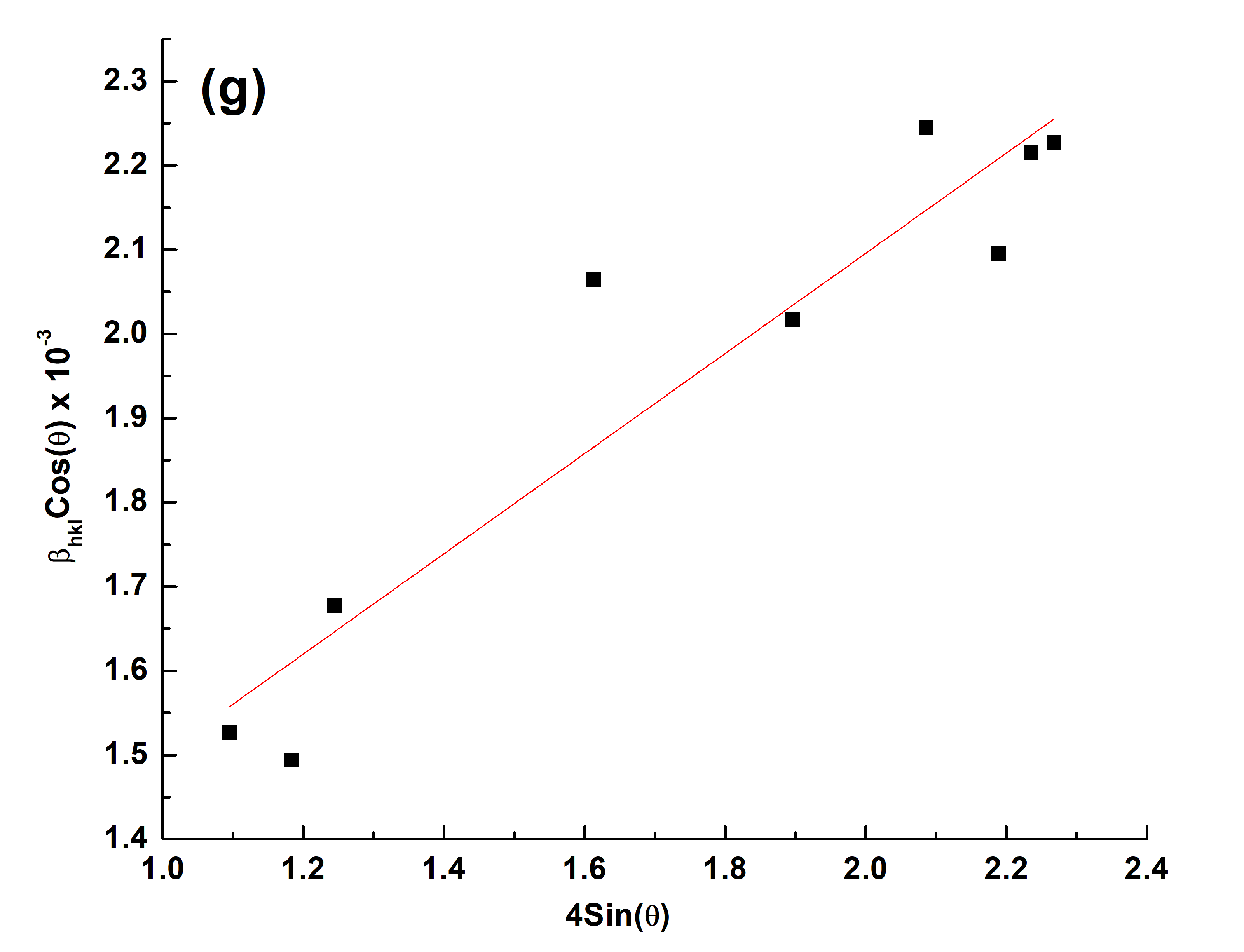 | 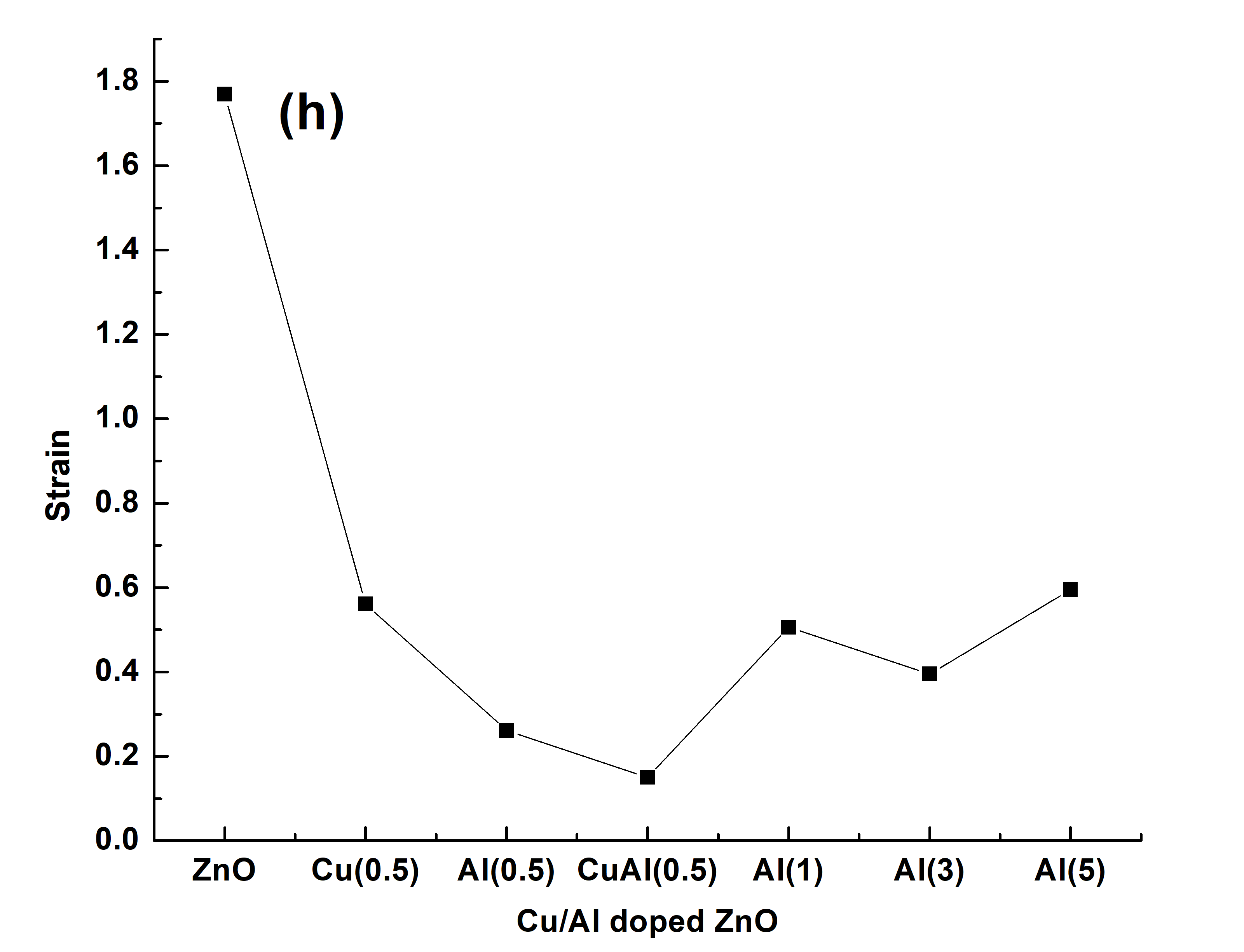 |

***Figure S14: W-H plots with linear fitting based on UDM for strain approximation (e) Al(1), (f) Al(3), (g) Al(5), and (h) behavior of strain value with increasing Al dopant .***

**Energy density analysis (W-H plots)**

| 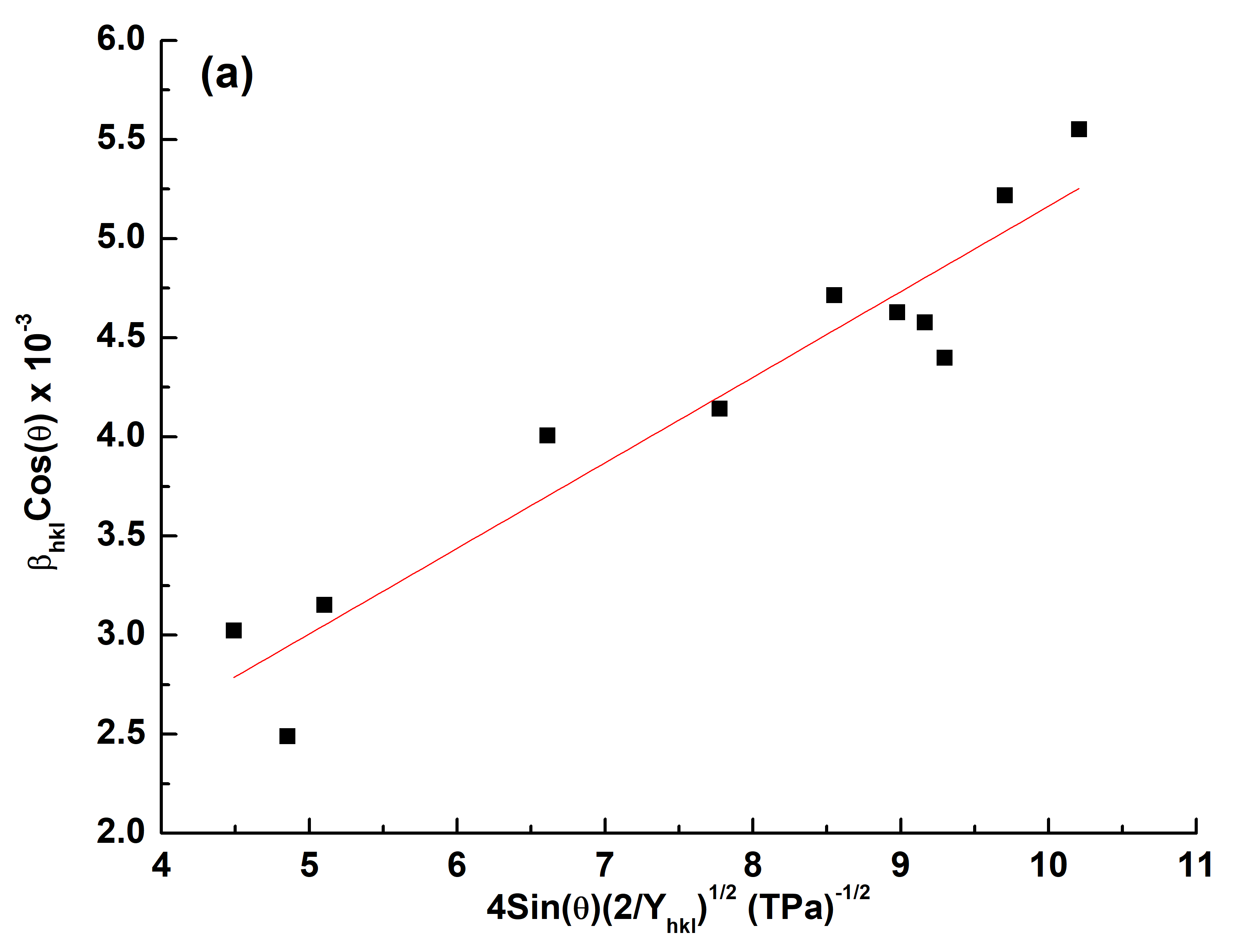 | 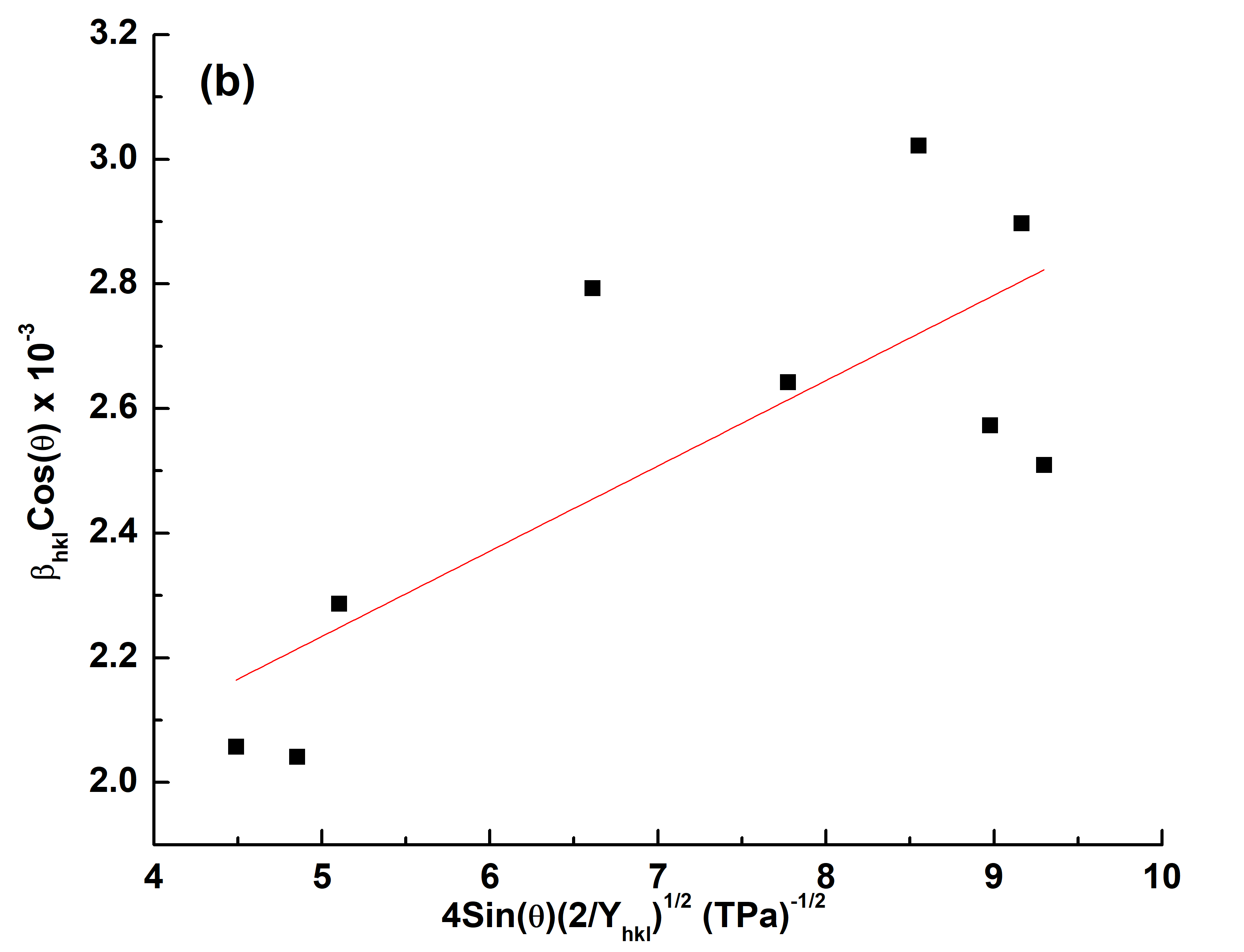 |
| --- | --- |
| 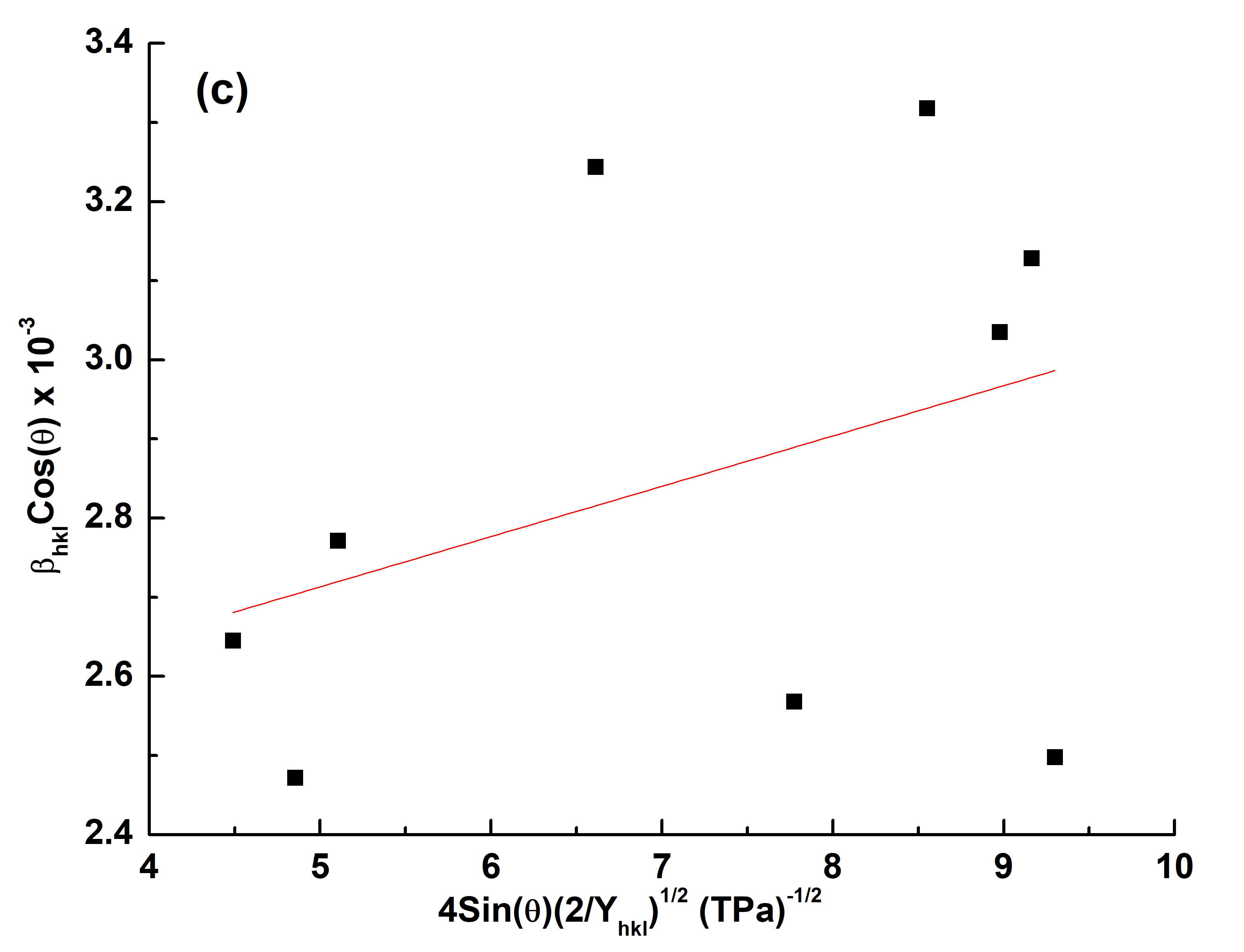 | 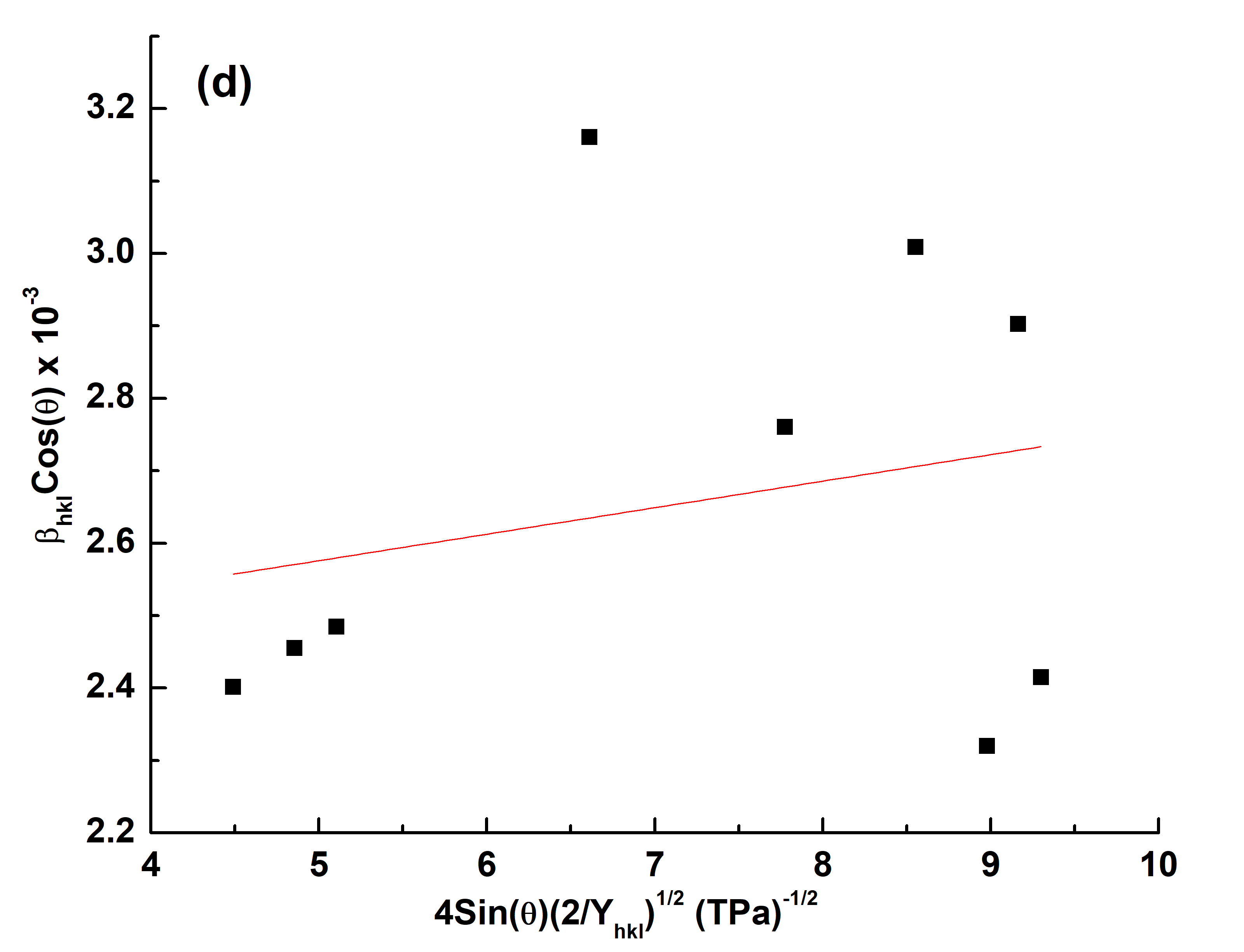 |

**Figure S15: W-H plots with linear fitting based on UDEDM for energy density a) pristine ZnO, (b) Cu(0.5), (c) Al(0.5), and (d) CuAl(0.5).**

| 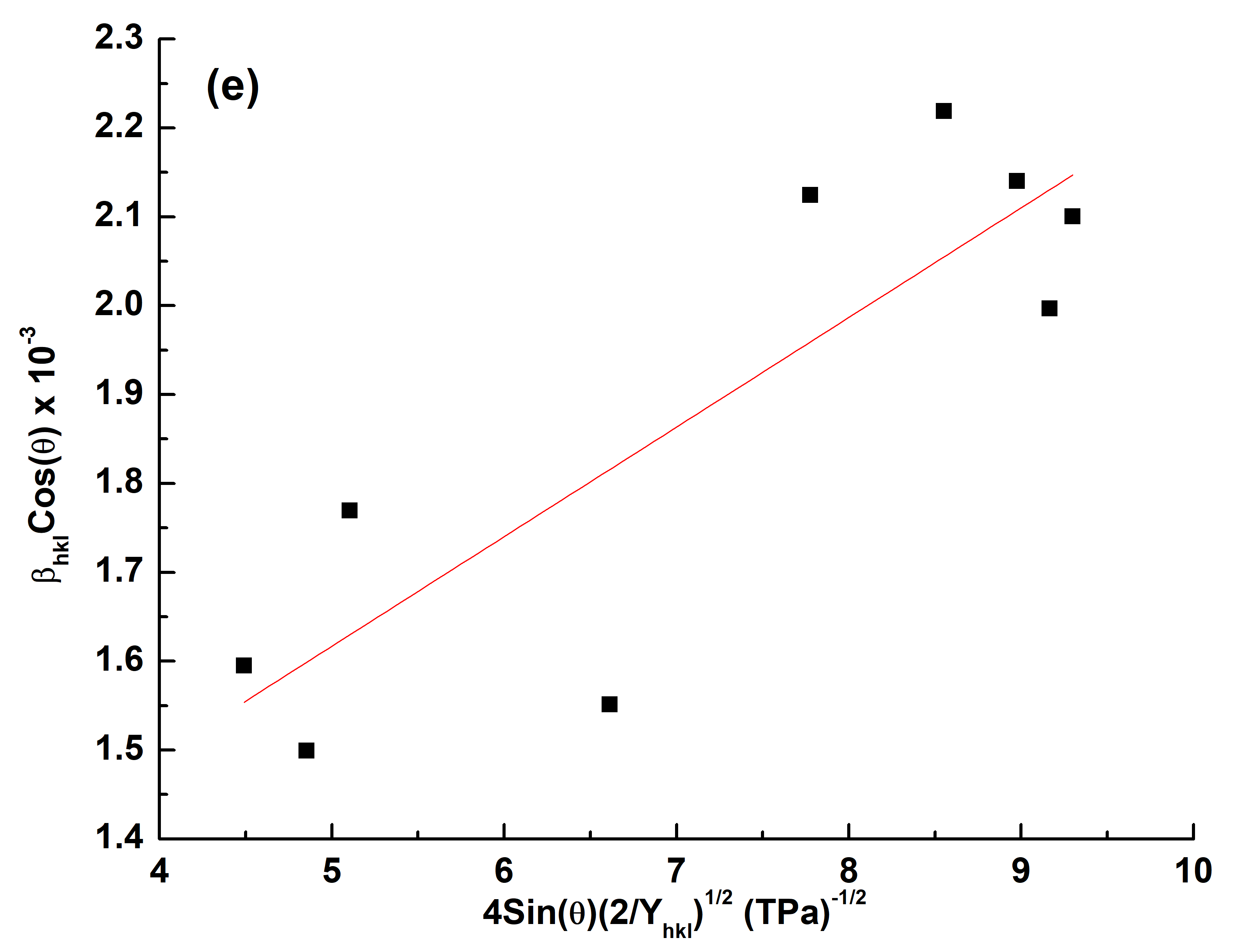 | 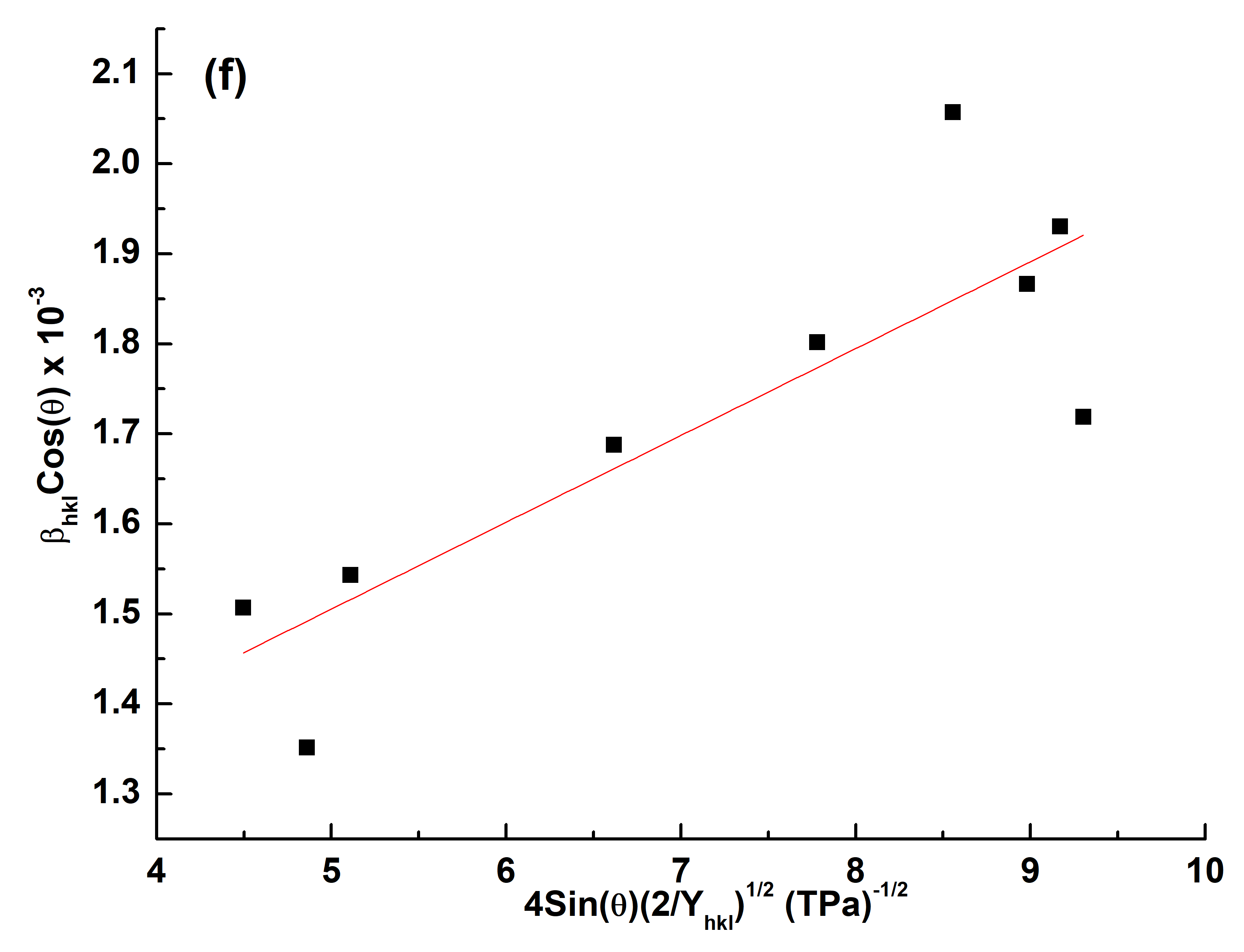 |
| --- | --- |
| 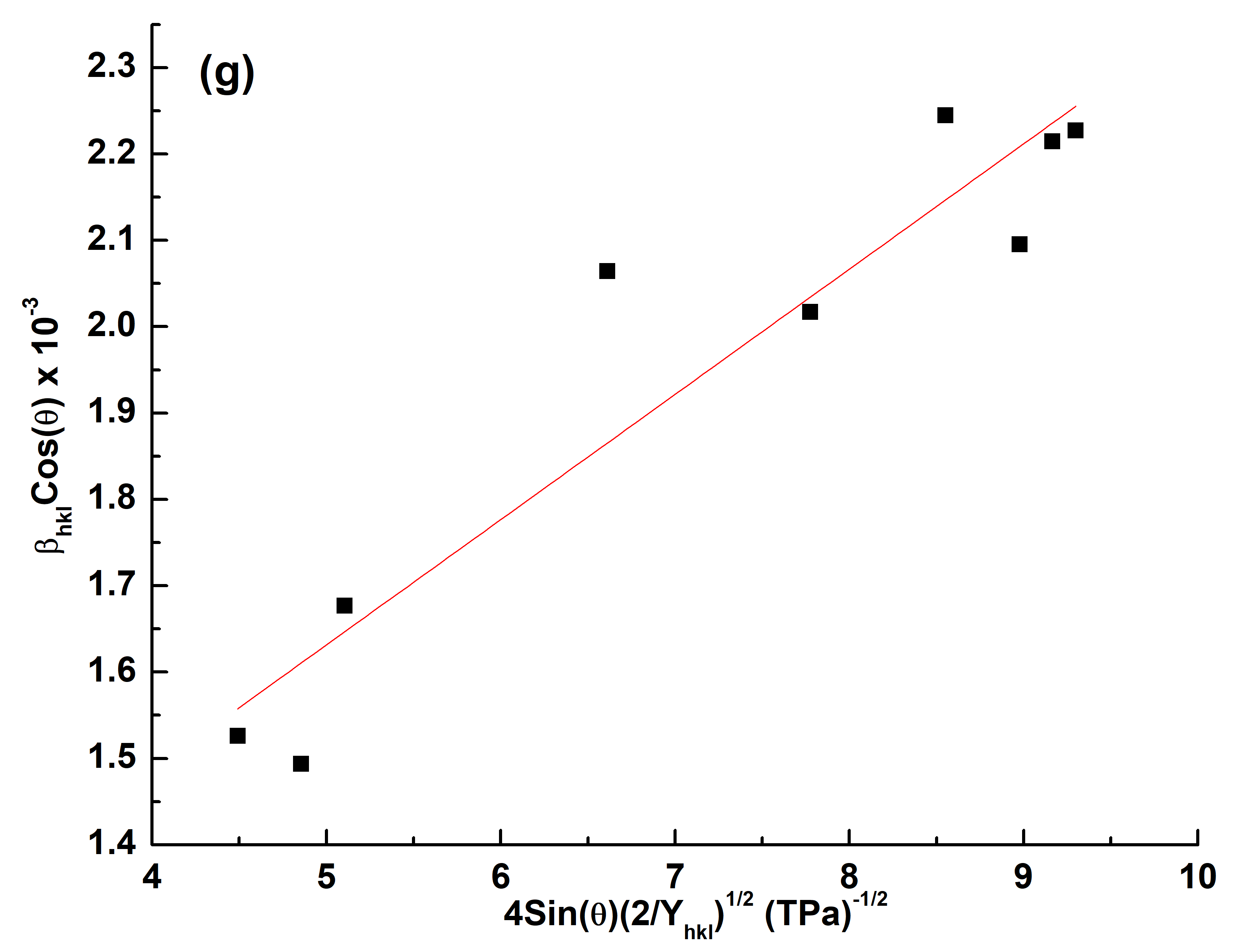 | 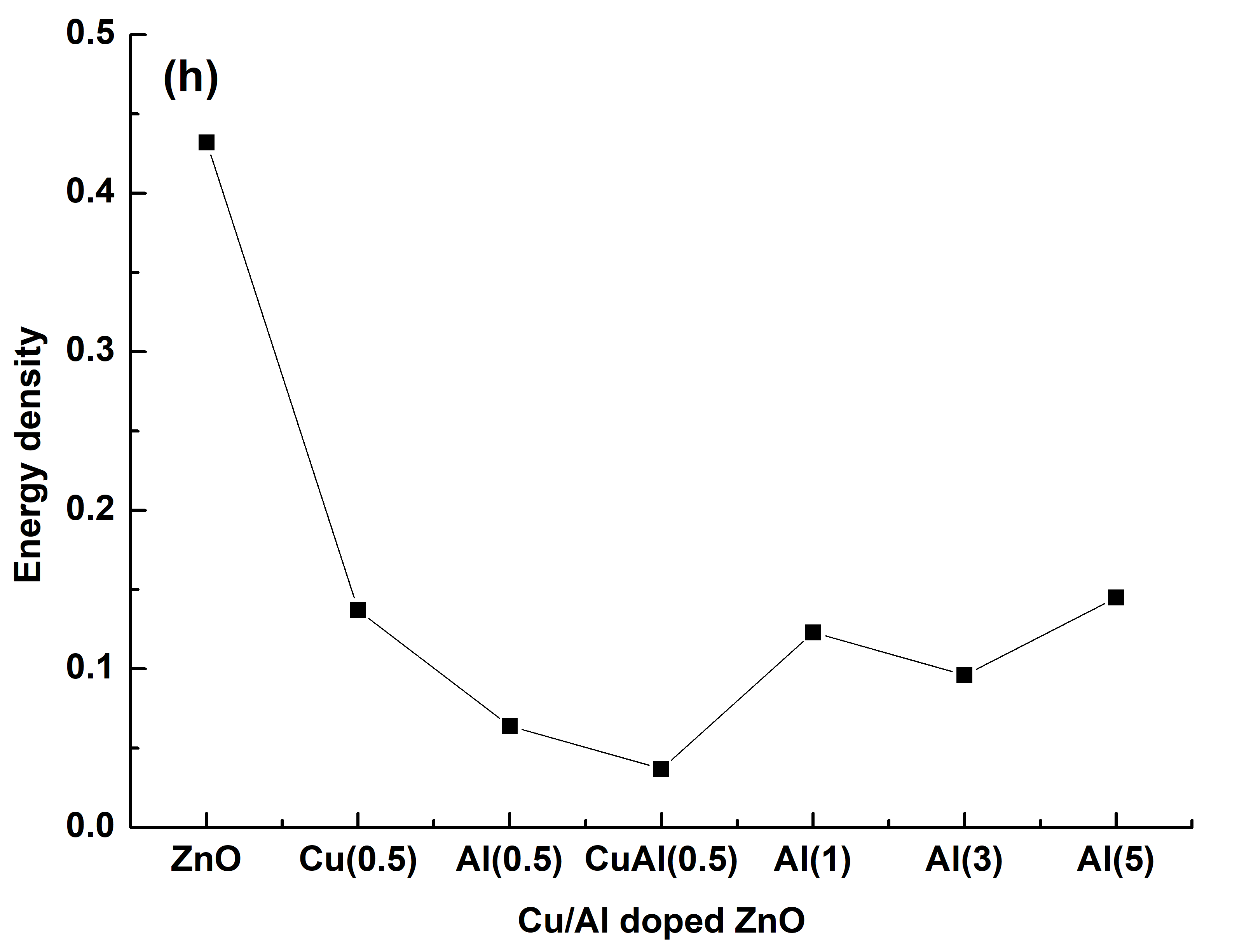 |

**Figure S16: W-H plots with linear fitting based on UDEDM for energy density (e) Al(1), (f) Al(3), (g) Al(5), and (h) behavior of energy density value with increasing Al dopant .**
